# Gene profiling of embryonic skeletal muscle lacking type I ryanodine receptor Ca^2+^ release channel

**DOI:** 10.1038/srep20050

**Published:** 2016-02-01

**Authors:** Dilyana Filipova, Anna M. Walter, John A. Gaspar, Anna Brunn, Nina F. Linde, Mostafa A. Ardestani, Martina Deckert, Jürgen Hescheler, Gabriele Pfitzer, Agapios Sachinidis, Symeon Papadopoulos

**Affiliations:** 1 Center of Physiology and Pathophysiology, Institute of Vegetative Physiology, Medical Faculty of the University of Cologne, Robert-Koch-Str. 39, Cologne 50931, Germany; 2 Center of Physiology and Pathophysiology, Institute of Neurophysiology, Medical Faculty of the University of Cologne, Robert-Koch-Str. 39, Cologne 50931, Germany; 3 Department of Neuropathology, University Hospital of Cologne, Cologne, Germany

## Abstract

In mature skeletal muscle, the intracellular Ca^2+^ concentration rises dramatically upon membrane depolarization, constituting the link between excitation and contraction. This process requires Ca^2+^ release from the sarcoplasmic reticulum via the type 1 ryanodine receptor (RYR1). However, RYR1’s potential roles in muscle development remain obscure. We used an established RyR1- null mouse model, *dyspedic*, to investigate the effects of the absence of a functional RYR1 and, consequently, the lack of RyR1-mediated Ca^2+^ signaling, during embryogenesis. Homozygous dyspedic mice die after birth and display small limbs and abnormal skeletal muscle organization. Skeletal muscles from front and hind limbs of dyspedic fetuses (day E18.5) were subjected to microarray analyses, revealing 318 differentially expressed genes. We observed altered expression of multiple transcription factors and members of key signaling pathways. Differential regulation was also observed for genes encoding contractile as well as muscle-specific structural proteins. Additional qRT-PCR analysis revealed altered mRNA levels of the canonical muscle regulatory factors *Six1, Six4, Pax7, MyoD, MyoG* and *MRF4* in mutant muscle, which is in line with the severe developmental retardation seen in dyspedic muscle histology analyses. Taken together, these findings suggest an important non-contractile role of RyR1 or RYR1-mediated Ca^2+^ signaling during muscle organ development.

Calcium is a key factor in a plethora of signaling pathways and cellular processes, including differentiation, growth, apoptosis, metabolism and transcriptional regulation. In developing skeletal muscle Ca^2+^ is required for myoblast migration, fusion and terminal differentiation, and for muscle growth^1^,^2^. Beyond this, Ca^2+^ is an essential regulator of muscle contraction^3^.

An important reservoir for changes in cytosolic calcium, [Ca^2+^]_i_, and by far the dominating source in differentiated skeletal muscle, is the sarcoplasmic reticulum (SR). The two most important Ca^2+^ channels for Ca^2+^ release from the internal stores during skeletal muscle development and differentiation appear to be the inositol 1,4,5*-*trisphosphate receptor (IP3R) and the type 1 ryanodine receptor (RyR1). On the basis of the kinetics of [Ca^2+^]_i_ transients, these two channels have been assigned slow (IP3R) and fast (RyR1) Ca^2+^ transients, respectively^4^. The fast Ca^2+^ transients are the typical stimulus for triggering muscle contraction via excitation-contraction coupling (ECC). In contrast to the fast mechanism, the slow Ca^2+^ transients consist of two kinetically discernible components, both characterized by subthreshold Ca^2+^ levels with respect to initiation of contraction. However, it has been demonstrated that the faster of the two slow components, termed “slow-rapid”, is also mediated by RyR1 and is prominent in both cytoplasm and nucleus, whereas the second, termed “slow-slow” is confined to the nucleus^5^ and is generated by Ca^2+^ release through the IP3R, which localizes to the nuclear envelope and also to distinct, extra-junctional regions of the SR^6^,^7^. Slow [Ca^2+^]_i_ kinetics have been linked to signaling^5^ via activation of the nuclear factor kappa B (NFκB) as well as the mitogen-activated protein kinase (MAPK) pathway through ERK1/2, CREB, and the early response genes *c-Jun* and *c-Fos*, in response to depolarization and to other stimuli like reactive oxygen species (ROS) and hormones, like insulin, for instance^8^,^9^,^10^. Thus, a crucial role of slow Ca^2+^ transients in muscle cells for downstream gene expression, and its relevance for skeletal muscle adaptation, becomes apparent. Different models, ranging from the C2C12 muscle cell line, primary muscle cell cultures at different differentiation states, to mouse muscle fibers, have been used to investigate the effects of [Ca^2+^]_i_ dynamics on gene expression. The usage of these diverse models has occasionally led to differing conclusions about the relative relevance of IP3R and RyR1-mediated Ca^2+^ release. However, even when using the same model the observations can occasionally differ, probably due to differences in experimental setup and conditions^11^. Currently, the relevance and the relative contribution of RyR1-mediated Ca^2+^ release in gene regulation during myogenesis and muscle differentiation is not clear.

The dyspedic mouse model, a RYR1-null mutant, has proven as a valuable model system for the investigation of ECC in skeletal muscle^12^,^13^. While heterozygous mice of the model are functionally indistinguishable from wild type (WT) littermates, homozygous dyspedic mice (referred to as *dysp* in the sequel) die at birth from asphyxia, due to paralysis of skeletal muscle including the diaphragm. Furthermore, homozygous mice are characterized by an abnormal spine curvature, subcutaneous hematomas, enlarged neck and small limbs^12^. The latter implicates a dysregulation of embryonic myogenesis in the *dysp* mutant. While a similar phenotype is reproduced by a mouse model carrying the central core disease mutation RyR1^I4895T^, which renders the RyR1 channel non-functional in terms of Ca^2+^ release^14^, no study has so far investigated the transcriptomic consequences of the lack of RyR1-mediated Ca^2+^ signaling during skeletal myogenesis. In order to elucidate manifested differences between *dysp* fetuses and their heterozygous control littermates, we used animals at stage E18.5. In addition to a macroscopic and microscopic morphology analysis, we extracted mRNA from front and hind limb muscles of four *dysp* and four control fetuses, and subjected it to microarray analyses (total of 8 microarrays). We identified more than 300 differentially expressed genes (DEGs), the expression of which was decreased or increased by at least 1.5-fold in *dysp* compared to their control littermates. Our results reveal an extensive downregulation of multiple DEGs encoding structural and contractile muscle proteins, and indicate alterations in extracellular matrix (ECM) composition. Moreover, the absence of the RYR1 protein and, consequently, RYR1-mediated Ca^2+^ release, in *dysp* muscle resulted in the transcriptional dysregulation of multiple members of major signaling networks like the MAPK pathway, the Wnt signaling pathway and the PI3K/AKT/mTOR pathway. A further analysis revealed significant differences in the mRNA levels of the key myogenic factors Six1, Six4, Pax7, MyoD, MyoG and Mrf4, corroborating the genetic basis for the delay in myogenesis.

Thus, our studies of on *dysp* skeletal muscle reveal extensive alterations in the transcriptional regulation of numerous genes coding for structural, metabolic and regulatory proteins, suggesting that RYR1-mediated Ca^2+^ release plays a pivotal role not only in muscle contraction, but also in the orchestration and coordination of skeletal muscle development and differentiation.

## Results

### Histological analysis of *dysp* limb skeletal muscle

**Figure 1:**
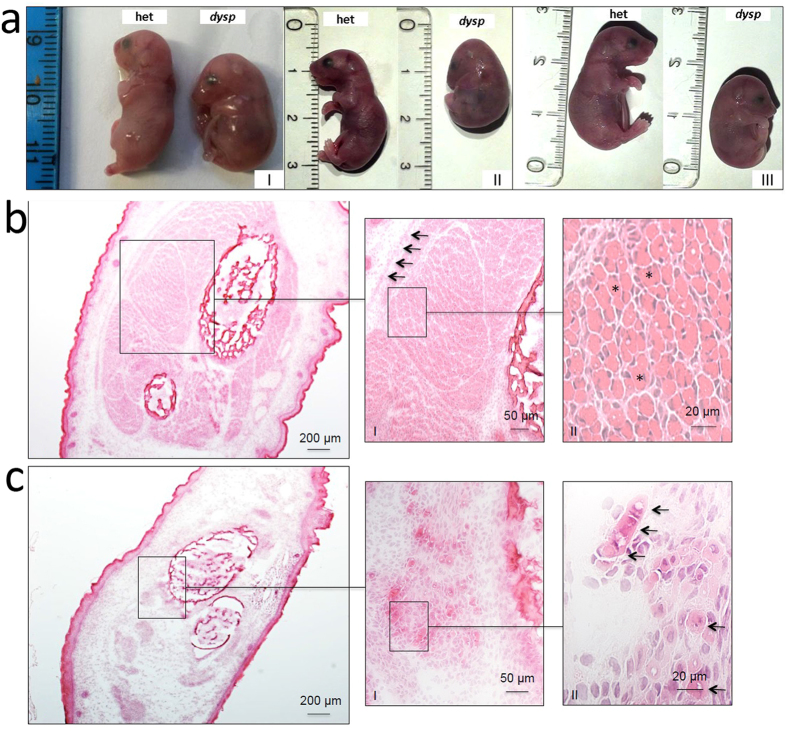
Developmental retardation and disorganization of skeletal muscle of *dysp* mice. (**a**) Heterozygous controls (het, left) and *dysp* (right) littermates at day E18.5 from three different litters (I, II and III). The *dysp* littermates display abnormal spine curvature, small limbs and enlarged necks. (**b**) At E18.5, the distal hind limb of control littermates contains well-developed muscle fibres (*) organized in fascicles, which are surrounded by an epimysial fascia (arrows). (**c**) In contrast, at E18.5 the distal hind limb of *dysp* mice contains only immature small fibers (arrows) in a scattered distribution, lacking a fascia (**b**,**c**): H&E staining; original magnification × 50 (**a**,**b**); × 200 (insets I); × 400 (insets II).

The histology of E18.5 skeletal muscle from homozygous *dysp* mice displayed severe disorganization and showed indications for developmental retardation (Fig. 1). In contrast to heterozygous mice of the same developmental stage, which had well developed muscles, organized in fascicles and covered by a fascia, skeletal muscle of *dysp* mice displayed only small groups of muscle cells, lacking organized fascicles and a fascia (Fig. 1b,c).

### Microarray analysis of *dysp* limb skeletal muscle

In an attempt to elucidate the basis for the drastic phenotypic alterations observed in *dysp* skeletal muscle, we performed microarray analyses of the transcriptome of fore- and hind limb skeletal muscle from four *dysp* and four control mouse fetuses at E18.5. The analyses returned 45,101 hits (identified transcript sets), spanning 21,569 unique annotated genetic loci. After data normalization and subsequent statistical analysis (see Materials and Methods) we identified 417 genomic loci, the expression of which was significantly (FDR-adjusted P value ≤ 0.05) positively or negatively regulated by at least 1.5-fold compared to the control (Supplementary Table S1). Of these, 394 mapped within known genes and 23 mapped at non-annotated genomic positions. The 394 differentially expressed loci were matched to 318 unique differentially expressed transcripts, of which 159 were positively regulated and 159 were negatively regulated in *dysp* skeletal muscle.

**Figure 2:**
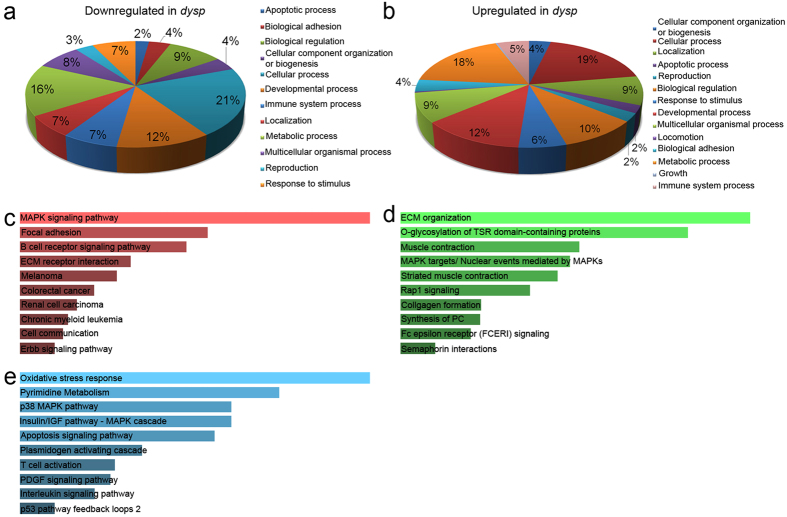
Functional classification of the 318 unique DEGs. (**a**) Downregulated (FC ≤ −1.5, P < 0.05) and (**b**) upregulated (FC ≥ 1.5, P < 0.05) genes were classified according to their involvement in biological processes, cellular components and molecular functions via *DAVID GO*^15^ and the 10 most significantly (P-value ≤ 0.05, Supplementary Table S2) regulated GO categories are represented as percentage of all genes from these categories. Genes not matching any of the classes are not considered in the above pie charts. All DEGs were subjected to an enrichment analysis via the online gene list analysis tool, *Enrichr*^16^, and were assigned to different pathways according to the KEGG (**c**), Reactome (**d**) and Panther (**e**) data bases. Bars in (**c**–**e**) are in the order of their P-value ranking.

The DEGs were classified into functional categories via the web-based Gene Ontology (GO) analysis tool *DAVID*^15^, using the groupings *biological process*, *cellular compartment* and *molecular function* (Fig. 2a,b; Supplementary Table S2). The 10 most significantly regulated GO categories, enriched with downregulated DEGs (Fig. 2a), contain multiple muscle-specific structures and processes, including *myofibril* (6 DEGs), *contractile fiber* (6 DEGs), *I band* (4 DEGs) and *muscle organ development* (6 DEGs). The GO enrichment analysis for upregulated DEGs (Fig. 2b) identified the regulation of *apoptosis/ programmed cell death* (16 DEGs) as the most significantly regulated GO categories. Interestingly, both induction (6 DEGs) as well as negative regulation (7 DEGs) of apoptosis were solidly identified.

In order to gain a better understanding about which molecular functions might have been affected by the transcriptomic alternations in *dysp* muscle, the 318 identified DEGs were subjected to a gene enrichment analysis according to the *KEGG, Reactome* and *Panther* data bases (Fig. 2c–e)^16^. Some of the well represented molecular functions and processes across the examined data bases are focal adhesion, ECM organization, ECM-receptor interaction, collagen formation and, most interestingly, muscle and striated muscle contraction. The *KEGG* pathway analysis revealed the MAPK pathway as the most significantly affected pathway (Fig. 2c). This pathway was also identified by the *Reactome Pathways* (Fig. 2d) and the *Panther* data bases (Fig. 2e), the latter detecting also the p38 and IGF-I/MAPK/ERK cascades. Oxidative stress response, apoptosis and the p53 pathway, well known for their connection to (and regulation by) the MAPK pathway, have also been listed by the *Panther* data base enrichment analysis^17^,^18^,^19^.

### Validation of the microarray data via principal component analysis (PCA) and via qRT-PCR

**Figure 3:**
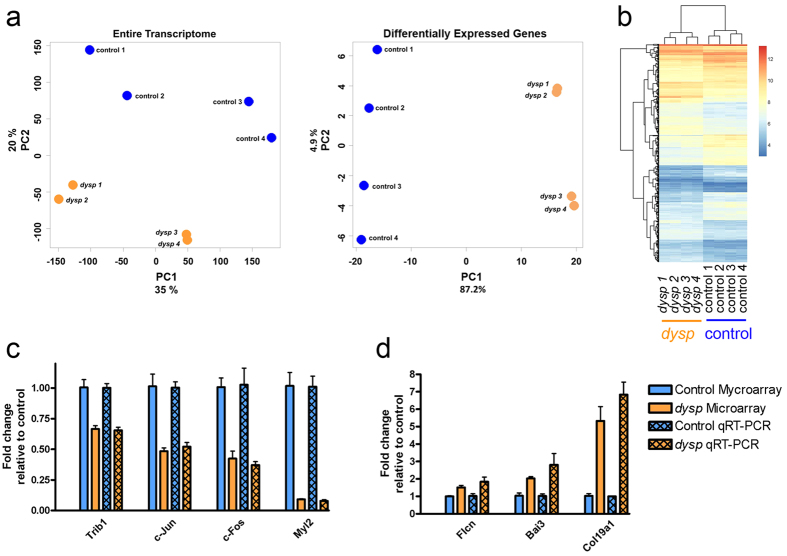
Microarray examination and validation via qRT-PCR (**a**) Principal component analysis (PCA) for all transcripts identified in the microarrays (left) and for the DEGs having a FC ≥ ±1.5 and a P value ≤ 0.05 (right). Each spot subsumes the DEGs of one biological replicate (four control animals, blue, four *dysp* samples, orange). (**b**) Heat map representing the log_2_ expression values for the DEGs having a FC ≥ ±1.5 and P value ≤ 0.05. Each column represents one biological replicate (columns 1 to 4 represent the *dysp* replicates and columns 5 to 8 represent the control group). (**c, d**) We selected 7 DEGs from the microarrays for re-examination via qRT-PCR: 4 downregulated genes with FCs −1.50 (*Trib1*), −2.07 (*c-Jun*), −2.43 (*c-Fos*) and −10.85 (*Myl2*) (**c**), as well as 3 upregulated genes with FCs 1.50 (*Flcn*), 2.02 (*Bai3*) and 5.13 (*Col19a1*) (**d**). *Gapdh* was used as an intrinsic reference. The mRNA levels are expressed as the mean FC of the 4 biological replicates of each group (*dysp* and control), normalized to the FC of the respective control, ±S.E.M.

In order to assess the variance between the *dysp* and control group, as well as between the biological replicates within each group, PCA was performed for all genes identified in the microarrays (Fig. 3a, left) as well as for the subset of 318 DEGs with fold changes (FC) ≥ ±1.5 and P values ≤ 0.05 (Fig. 3a, right). Each spot in Fig. 3a represents one biological replicate. The PCA plot generated for all transcripts shows a clear grouping of the *dysp* and control samples. The PCA plot generated for the 318 DEGs demonstrates a strong correlation between the intra-group replicates (*dysp* vs. *dysp*; control vs. control) compared to inter-group replicates(*dysp* vs. control), as the principal component (PC) 1 is showing a 87.2% variance while in PC 2 this value only amounts to 4.9%. A heat map representation of the 318 DEGs of each biological replicate is shown in (Fig. 3b).

Selected genes, for which our microarray analyses had reported changes in their expression, were validated via real-time quantitative PCR (qRT-PCR). The genes chosen include both downregulated (Fig. 3c) as well as upregulated (Fig. 3d) species, with strong as well as weak fold changes (FC). For all of the analyzed DEGs, qRT-PCR results confirmed our microarray data with respect to both, direction of change (up- or downregulation) and degree of change in expression, FC.

### Transcripts displaying the highest fold-changes in *dysp* skeletal muscle

The ten genes with the highest fold-change (in positive as well as in negative direction) we found in our microarray analysis are shown in Table 1. Highest induction (6.5-fold) was observed for collagen type XXV alpha 1 (*Col25a1*) and similarly for another collagen, type XIX alpha 1 (*Col19a1*), implicated in early myogenesis^20^. Among the downregulated genes in *dysp* muscle, the gene encoding myosin light chain 2 (*Myl2*) showed the lowest expression rate (−10.8-fold). Table 1 also lists several other important genes, associated with muscle structure and function, like *Tppp3*, *Irf6* and *Cnn1*. These latter genes were negatively regulated to a high degree (Table 1).

### Signaling pathways enriched with DEGs in *dysp* skeletal muscle

Our analysis identified several major signaling pathways as being substantially enriched with DEGs. In particular, the MAPK pathway is represented with 21 DEGs, encoding proteins involved at different stages of Ras, JNK and p38 signaling (Table 2), 7 of which are positively and 14 negatively regulated. Interestingly, the majority of downregulated DEGs encode proteins that engage late downstream in the pathway, like the FBJ osteosarcoma oncogene (*c-Fos*), the Jun oncogene (*c-Jun*), the Jun proto-oncogene related gene D (*Jund*) and the calcineurin dependent nuclear factor of activated T cells 2 (*Nfatc2*). These genes encode global transcription factors that regulate transcription in response to various stimuli, modulating a variety of cellular responses and processes, including proliferation, differentiation, inflammation and apoptosis. Notably, the *c-Fos, c-Jun* and *Jund* genes all encode different dimerization partners within the composition of the pleiotropic transcription factor activating protein-1 (AP-1). Another interesting finding is that several DEGs encoding cell surface receptor proteins like the neurotrophic tyrosine kinase receptor type 2 (*Ntrk2*), the transforming growth factor beta receptor I (*Tgfbr1*), as well as the beta 4 subunit of voltage-dependent calcium channels (*Cacnb4*), all linked to the MAPK pathway, were upregulated. We observed a negative regulation of four dual specificity phosphatase transcripts (*Dusp1, Dusp8, Dusp10 and Dusp16*), as well as the heat shock protein 1-like (*Hspa1l*) gene, all of which inactivate ERK, JNK or p38. Additionally, among the DEGs of the MAPK pathway, there are several genes encoding key proteins connecting multiple signaling pathways. For example, the thymoma viral proto-oncogene 2 (*Akt2*), found to be positively regulated by our microarray analyses, encodes a central member of the PI3K/Akt/mTOR pathway, also represented with several other DEGs, including the Akt’s activator, the p85 alpha regulatory subunit of the phosphatidylinositol 3-kinase (*Pik3r1*) and Akt’s target, the gene encoding the cyclin-dependent kinase inhibitor 1A (P21) (*Cdkn1a*). 10 DEGs are associated with the Wnt signaling pathway, including the downregulated genes wingless-related MMTV integration site 2 (*Wnt2*), secreted frizzled-related protein 4 (*Sfrp4*) and the induced transcript 1 of transforming growth factor beta 1 (*Tgfb1i1*), as well as secreted frizzled-related protein 1 (*Sfrp1*), one of the few positively regulated transcripts in this pathway. Many of the identified DEGs encode G protein coupled receptors (GPCRs) or modulators of GPCR-mediated signaling, as well as various transcription factors. Thus, the microarray analysis indicates substantial changes in the expression profile of global signaling networks. Several of the DEGs involved in ubiquitous signaling pathways have been previously linked to muscular processes and are described later in this manuscript in the context to of muscle development and function.

### Muscle specific processes enriched with DEGs in *dysp* skeletal muscle

Enrichment analyses (Fig. 2) revealed a strong change in the transcription levels of genes involved in muscle contraction as well as in processes like focal adhesion, ECM organization, ECM-receptor interaction and collagen matrix formation. These processes are intimately linked to the development and morphogenic structure of the skeletal muscle organ. In order to further analyze which of the 318 DEGs are particularly involved in processes related to muscle development and function, we applied the online enrichment tools *DAVID GO, Panther GO and MGI GO*, combined with manual data mining. In doing so, we identified 21 genes (16 downregulated and 5 upregulated) unambiguously related to muscle force production and to the components of the contraction apparatus (Table 3 a). Specifically, these genes encode sarcomeric and costameric proteins, as well as proteins involved in excitation-dependent processes.

We furthermore identified 22 genes (14 downregulated and 8 upregulated) related to mostly structural features of the muscle organ, comprising proteins of the extracellular matrix, the cytoskeleton and transmembrane/cell-surface proteins (Table 3 b).

### Altered expression of myogenic regulatory factors in *dysp* skeletal muscle

**Figure 4:**
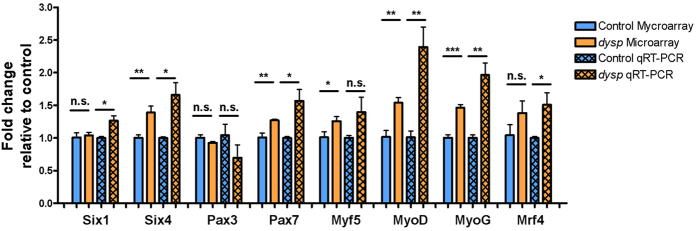
Comparison of mRNA levels of key myogenic regulatory factors between control and *dysp* skeletal muscle. Displayed are mRNA levels of the regulatory factors *Six1, Six4. Pax3, Pax7, Myf5, MyoD, MyoG* and *Mrf4*, as determined by microarray analyses and via qRT-PCR. Four biological replicates (4x control, 4x *dysp;* 8 animals in total) were run for every gene. In the qRT-PCR analyses, the respective *Gapdh* mRNA levels served as endogenous reference. The values on the ordinate emanate from normalizing the FCs of the 4 biological replicates (control or *dysp*) to the mean of the respective control. Thus, all controls amount to a “Fold change” of 1. Unpaired t-tests were performed for control vs. *dysp* for each gene, *represents a P value ≤ 0.05, **represents a P value ≤ 0.01 and ***represents a P value ≤ 0.001. Error bars are S.E.M.

Proper embryonic development of skeletal muscle is governed by both intrinsic and extrinsic mechanisms^21^. Among the most important intrinsic signals controlling myogenesis progression are the transcription factors Six1, Six4, Pax3, Pax7, MyoD, MyoG and Mrf4. Each of these is expressed in a specific population of cells from the myogenic lineage and has a discrete temporal expression pattern during myogenesis. While none of these factors passed our stringent threshold for being classified as significantly regulated (FC ≥ ±1.5, FDR-adjusted P value ≤ 0.05), the microarrays indicated changes in the expression levels of *Six4* (FC = 1.38, P = 0.0027), *Pax7* (FC = 1.27, P = 0.0074), *Myf5* (FC = 1.25, P = 0.04), *MyoD* (FC = 1.53, P = 0.0018) and *MyoG* (FC = 1.46, P = 0.0001) (Fig. 4). Moreover, the severe *dysp* histology shown in Fig. 1 suggested developmental defects, prompting us to closer examine the relative expression levels of these important developmental markers. qRT-PCR analysis revealed a significant (P ≤ 0.05) upregulation in the expression of *Six1, Six4, Pax7, MyoD, MyoG and Mrf4* in *dysp* muscle, with FCs 1.27 ± 0.07 (P = 0.0138) for *Six1*; 1.66 ± 0.19 (P = 0.0136) for *Six4*; 1.57 ± 0.18 (P = 0.0183) for *Pax7*; 2.39 ± 0.30 (P = 0.0049) for *MyoD*; 1.97 ± 0.18 (P = 0.0022) for *MyoG* and 1.51 ± 0.19 (P = 0.0343) for *MRF4* (Fig. 4). The increased transcript levels of myogenic markers in E18.5 dysgenic muscle are in line with the presence of a delay in myogenesis^21^.

## Discussion

The RYR1 Ca^2+^ release channel is a major component of the ECC apparatus in skeletal muscle and various mutations in its gene have been associated with a number of muscular disorders including malignant hyperthermia and several congenital myopathies^22^. The absence of RYR1 in homozygous *dysp* mice leads to excitation-contraction uncoupling and perinatal lethality, accompanied by abnormal alterations in muscle phenotype, indicative of impaired muscle development^12^. The goal of this study was to identify signaling pathways and biological processes influenced by RYR1 during skeletal muscle formation, at late stages of embryonic development. For this purpose, we performed a microarray analysis of limb skeletal muscle from *dysp* fetuses and their control litter mates at day E18.5. This approach led to the identification of more than 300 differentially expressed genes.

*Dysp* muscle lacks both, Ca^2+^ release for mechanical movement and RyR1-mediated Ca^2+^ signaling. It is likely the combination of both, ablation of mechanotransduction and of RyR1 dependent Ca^2+^ signaling, which leads to the severe phenotype of E18.5 *dysp* muscle. Although our data refer to embryonic muscle, we find parallels in the direction of transcriptional changes to animal models for acute denervation, amyotrophic lateral sclerosis (ALS) or toxin-induced paralysis. For instance, in *dysp* skeletal muscle we observe mRNA upregulation for the transcription factor Runx1, which has been implicated in the counteraction of muscle wasting, autophagy and myofibrillar disorganization^23^,^24^. Classically, we observe an upregulation of the acetylcholine receptor α (*Chrna1*) as well as the fetal γ subunit (*Chrng*)^25^, and of the acetylcholine receptor-associated protein of the synapse (*Rapsn*)^26^. Furthermore we see a downregulation for the ECM transcripts tenascin C (*Tnc*) and microfibrillar associated protein 5 (*Mfap5*)^27^,^28^ as well as a strong upregulation for collagen type XIXα1 (*Col19a1*), which has also been found to be upregulated in ALS muscle^29^.

However, some transcriptional changes in the *dysp* model contrast those seen in models for paralysis and denervation. For *Ankrd1*, a member of the titin-N2A mechanosensory complex of the Z-disc^30^ with roles in muscle morphogenesis and remodeling, we find a strong downregulation, whereas an upregulation was observed in ALS muscle and after denervation^23^,^29^ [noticeably, no *Ankdr1* at all was detected in a study of central core disease (CCD) in humans, a disease linked to deficient function of RyR1 Ca^2+^ release^31^]. The *Tnfrs12a* transcript, coding for the Tweak receptor, was shown to be upregulated upon denervation^32^ but we find it downregulated in *dysp* skeletal muscle. Both, Tweak and Ankrd1 are Bcl-3 targets and represent repression targets of p38γ^33^ (discussed below).

Interestingly, the “resting” [Ca^2+^]_i_ is ~2-fold lower in *dysp* compared to WT myotubes^34^. This already could have effects on Ca^2+^ dependent signaling. The consistent downregulation of *c-Fos*, *c-Jun*, *Jund* and *Nfatc2*, encoding phosphorylation targets of ERK1/2, JNK and p38, could imply a disturbed regulation of these central MAPKs, which are activated in a Ca^2+^-dependent manner^35^,^36^ (however, in intact muscle, all of the latter would be activated by mechanical stress as well and could exert their important roles in myogenesis^37^). We identified a number of DEGs, which, in the context of metabolic adaptation to exercise, are influenced by p38γ^33^. Thus, altered p38y activity in *dysp* muscle could affect oxidative metabolism. This notion is supported by the changes in transcription we see for various genes whose products take part in oxidative reactions, like the gene encoding methionine sulfoxide reductase B3 (*Msrb3*), or genes, the expression of which is regulated in response to oxidative stress, like thrombospondin 4 (*Thbs4*). The latter is expressed in red skeletal muscle, i.e. in fibers with high oxidative capacity^38^. However, we find it 2-fold downregulated in *dysp* skeletal muscle. Moreover, the expression of *c-Jun* and *c-Fos*, which has been shown to be positively regulated by oxidative stress in a RYR1-dependent manner^9^, is decreased in *dysp* muscle, indicating that the absence of RYR1 may also affect ROS-sensitive signaling cascades.

The formation of skeletal muscle is critically modulated by Wnt signaling^39^. Our results reveal a downregulation of Wnt signaling factors *Wnt2, Cd44, Fzd10, Tgfb1i1*, combined with the upregulation of *Sfrp1*, an inhibitor of Wnt signaling. Overall, in *dysp* muscle we see indications for a shift from canonical, pro-myogenic Wnt signaling to the less defined non-canonical pathway^40^. However, since non-canonical Wnt signaling can activate Ca^2+^ release via IP3R, this shift might represent a compensatory attempt to raise [Ca^2+^]_i_ in *dysp* myotubes. Moreover, the non-canonical pathway also appears to activate the Akt/mTOR pathway, involved in increased anabolic activity and in hypertrophy of skeletal muscle^41^. Accordingly, we observe an upregulation of the central (*Pik3r1, Akt2*) and late (*Cdkn1a*) stages of Akt/mTOR signaling.

Genes encoding structural proteins of muscle are also affected in *dysp* muscle. Previous studies indicated that the *dysp* skeletal muscle expresses the major elements of the triad junction^12^,^42^. However, the transcripts of many genes taking part in the formation, organization and structure of the muscle contractile apparatus (*Myl2, Myl3, Myl9, Smtln1, Cnn1, Tpm3, Ankrd1, Nrap, Csrp3, Pdlim1, Fhl1, Nes* and *Tnnt2*) are, with the exception of *Tnnt2*, negatively regulated (Table 1). Notably, many other negatively regulated DEGs (*Ankrd1, Nrap, Csrp3, Pdlim1, Fhl1, Nes*) encode proteins that localize to the Z disc of sarcomeres and some of them connect sarcomeres to the t-tubular system of the sarcolemma and to the SR. Although the above proteins are primarily known for their structural and contractile function, many of them also have important roles in mechanosensing and in signaling into gene expression^43^ and myogenesis. Thus, absence of RYR1 affects different levels of muscle cell function.

We also find transcriptional changes in genes encoding extracellular matrix (ECM) proteins. *Mfap5, Tnxb, Tnc, Fn1, Adamtsl4* and *Fbn1* are involved in microfibrillar assembly and in matrix structuring, and their changes suggest an impaired elastic fiber formation^44^,^45^,^46^,^47^. Conversely, the expression level of three collagen species (*Col20a1, Col19a1* and *Col25a1*) is strongly upregulated in *dysp* muscle (Table 1). These changes may indicate a shift in the ECM composition towards collagen fibers at the expense of elastic fibers. Since mechanical loading is a critical stimulus in organization and turnover of ECM in skeletal muscle^48^, the immobilization in *dysp* skeletal muscle might contribute to the observed tissue disorganization (Fig. 1b,c).

Although their extensive discussion is beyond the scope of our study, we should mention that genes associated with other important components or processes of skeletal muscle were also among the DEGs of *dysp* muscle, like satellite cells (*Six1*, *Six4*, *Pax7*, *Sfrp4*, *Dusp10*, *Nes, Rgs5*, *Cav2*, *Megf10*, *Hgf*, *Ptpz1*, *Aif1*, *Cnr1*), myoblast fusion and differentiation (*Mfap5*, *Nov*, *Dpysl3*, *Wnt2*, *Cd44*, *Nfatc2*, *Cdkn1a*, *Hes6*, *Akt2*, *Adamtsl2*, *Hdac4*, *Fst*, *Sfrp1*, *Bai3*, *Marveld2*), and terminal muscle differentiation (*Myod*, *Myog*, *Mrf4*, *Hes6*, *Csrp3*,*Bcl6*, *Fgf6*, *Nfatc2*) (Supplementary Table 1). However, the observed upregulation of the canonical myogenic regulatory factors *Six1, Six4, Pax7, MyoD, Myog* and *MRF4*, the expression of all of which (except for *MRF4*) is attenuated in terminally differentiated, intact skeletal muscle^21^, already indicates that the virtually the entire developmental repertoire of myogenic factors is challenged in the *dysp* phenotype.

In conclusion, our report provides the first extensive skeletal muscle transcriptome analysis of the dyspedic mouse model, revealing that absence of the major Ca^2+^ release channel, RyR1, introduces multilayered transcriptomic alterations in developing skeletal muscle. The differential expression of genes, encoding a multitude of signaling and structural proteins important for embryonic development, suggests a complex regulatory role for RYR1 in myogenesis. This is reflected by the severely disorganized and developmentally retarded skeletal muscle histology, documenting the severe consequences of RyR1 absence. Further studies will aim to elucidate the exact molecular mechanisms of RYR1-mediated regulation of muscle organ development.

## Methods

### Animals and skeletal muscle preparation

Experiments with mice were carried out in accordance with the guidelines of the European Commission (Directive 2010/63/EU) and of the German animal welfare act (TierSchG). The mice were kept in the Animal Facility of the Medical Faculty of the University of Cologne according to the European Union Recommendation 2007/526/EG. All experimental protocols were approved by the local governmental authorities (Landesamt für Natur, Umwelt und Verbraucherschutz, North Rhine-Westphalia, AZ84-02.05.20.13.010). The dyspedic mouse line ry1^42^ with the background C57BL/6J was obtained from Vincenzo Sorrentino, University of Siena, upon generous approval by Paul Allen, UC Davis. Two heterozygous *dysp* male and female mice were subjected to timed mating. The pregnant females were sacrificed at day 18.5 post coitum by cervical dislocation. Two homozygous *dysp* and two control-pup littermates (as confirmed by subsequent PCR) from each female were prepared as described previously^49^ and used in the subsequent analyses (Supplemental Fig. 1). Skeletal muscle was dissected from the front and hind limb of the pups, pooled for each animal, frozen and stored in liquid nitrogen until use. The samples from each animal were collected and treated separately for all subsequent analyses, yielding 4 biological replicates per group (*dysp* and control), respectively.

### Morphological analysis

To give an impression of how *dysp* fetuses present at E18.5 in comparison to their unaffected heterozygous littermates, the body shape and size was documented by photographs. Horizontal and longitudinal sections of the entire front and hind limbs of E18.5 weeks old *dysp* fetuses and their WT littermates were prepared and mounted on thick filter paper with Tissue Tek OCT compound (Miles Scientific, Naperville, IL), snap-frozen in isopentane (Fluka, Neu-Ulm, Germany) pre-cooled by dry ice, and stored at −80 °C until preparation of serial 10-μm frozen sections. Enzyme histochemistry was performed with reactions for myofibrillar ATPase at pH 9.4 and pH 4.6, acid phosphatase, oil red, and reduced nicotinamide adenine dinucleotide-tetrazolium reductase (NADH).

### RNA isolation and purification

For RNA extraction, the muscle tissue was homogenized mechanically via a steel micropestle (Cat. #6-1062, neoLab, Heidelberg, Germany) in liquid nitrogen. Total RNA was extracted via the *Maxwell 16 LEV simplyRNA Tissue Kit* (Cat. #AS1280, Promega, Madison, WI) on a Maxwell 16 Instrument (Cat. #AS2000, Promega, Madison, WI) according to the manufacturer’s instructions. RNA concentration was measured with a NanoDrop 1000 Spectrophotometer (Peqlab, Erlangen, Germany).

### Microarray

All reagents and instrumentation pertaining to oligonucleotide microarrays were purchased from Affymetrix (Affymetrix, Santa Clara, CA, USA, http://www.affymetrix.com). Total RNA (100 ng) was used for amplification and *in-vitro* transcription using the Genechip 3′ IVT Express Kit as per the manufacturer’s instructions (Affymetrix). The amplified RNA was purified with magnetic beads and 15 μg Biotin-aRNA was fragmented with fragmentation reagent. 12.5 μg of fragmented aRNA was hybridized to Affymetrix Mouse Genome 430 2.0 arrays along with hybridization cocktail solution and then placed in Genechip Hybridization Oven-645 (Affymetrix) rotating at 60 rpm at 45 °C for 16 h. After the incubation arrays were washed on a Genechip Fluidics Station-450 (Affymetrix) and stained with Affymetrix HWS kit as per manufacturer’s protocols. The chips were scanned with Affymetrix Gene-Chip Scanner-3000-7G and the quality control matrices were confirmed with Affymetrix GCOS software following the manufacturer’s guidelines.

### Statistical analysis and identification of differentially expressed genes

Robust Multi-array Analysis was used for background correction, summarization and normalization^50^. The quantile normalization method was implemented to normalize the raw dataset executable with R-package^51^, carried out at the probe feature level. The differentially expressed genes were described by a linear model implementing R and the LIMMA packages^52^. Differentially expressed genes were determined based on cut-off values of 5% error rate (P < 0.05), calculated by moderated t- statistics according to Benjamini and Hochberg (Multiple Testing Correction)^53^. Additionally, to identify significantly expressed genes between the control and *dysp* sample groups, the size of change with the threshold value ≥ ±1.5 was used. Principal component (PC) analysis was performed using the Stats package in R using the prcomp function. The “x” attribute of the prcomp object was used to generate 2 dimensional scatter plots. Microarray data are available in the ArrayExpress database (www.ebi.ac.uk/arrayexpress) under the accession number E-MTAB-3608.

### Gene Ontology Enrichment Analysis

Gene Ontology analysis for the list of differentially expressed genes was performed to identify their prevalence in Biological processes and in molecular functions and pathways, with the help of the *DAVID* (Database for Annotation, Visualization and Integrated Discovery, http://david.abcc.ncifcrf.gov/)^15^, MGI^54^ and Enrichr^16^ functional annotation tools, with the Fisher Exact P- value set to <0.01.

### cDNA synthesis and qRT-PCRs

100 ng total RNA of each sample were used for cDNA synthesis via the “First Strand cDNA Synthesis Kit” (Cat. #E6550, New England Biolabs, Ipswich, MA) according to the manufacturer’s instructions. qRT-PCR was used for determination of the relative gene expression changes of selected genes of interest. All primers (Table 4) were designed using the OligoPerfect™ Designer (Life Technologies) with a T_m_ range of 58 °C–60 °C , an optimal length of 20 bases and an amplicon of 100–120 bp, and were purchased from Sigma Aldrich. The qRT-PCR reaction mixtures were mixed in 0.1 ml MicroAmp Fast 96-well Reaction Plates (Cat. #4346907, life Technologies, Darmstadt, Germany). The GoTaq® qPCR Master Mix kit (Cat. #A6001, Promega, Madison, WI) was used for preparation of the reaction mixtures according to the manufacturer’s instructions in a final volume of 20 μl per reaction. cDNAs were diluted 1:10 with RNase-free water and 4 μl of the dilutions were used as a reaction template. qRT-PCRs were performed in a thermo-cycler (StepOnePlus™ real-time PCR System, life Technologies, Darmstadt, Germany). Triplicates of each sample were assayed in one run (50 cycles) composed of three stages: 1. Activation at 95 °C for 10 min, 2. Denaturation at 95 °C for 15 s and annealing/extension at 60 °C for 1 min for each cycle, 3. Melt curve at 95 °C for 15 s, 60 °C for 1 min and 95 °C for 15 s.

qRT-PCR data were analyzed using relative quantification and the Ct method as described previously^55^, with the *Gapdh* gene as the endogenous control. The level of gene expression was calculated as ΔCT by subtracting the averaged Ct values (Ct refers to the threshold cycle) for *Gapdh* from those for the gene of interest. The difference in expression (*dysp* vs. control) was calculated as ΔΔCt. The relative expression of genes of interest was calculated and expressed as FC, 2^−ΔΔCt^. Bars in Figs 3 and 4 are represented as FCs plus/minus the standard error of the mean (S.E.M.) relative to the control group, which was normalized to an expression rate of 1. For each gene the expression levels of the *dysp* and control samples were subjected to an unpaired t-test and expression rates were assumed to be statistically significant upon a P value ≤ 0.05.

## Additional Information

**How to cite this article**: Filipova, D. *et al.* Gene profiling of embryonic skeletal muscle lacking type I ryanodine receptor Ca^2+^ release channel. *Sci. Rep.* **6**, 20050; doi: 10.1038/srep20050 (2016).

## Supplementary Material

Supplementary Information

## Figures and Tables

**Table 1:** The 10 strongest upregulated and downregulated probe sets identified in the microarray analysis.

Probe Set ID	Gene Title	Gene Symbol	FC
Downregulated genes			
1448394_at	myosin, light polypeptide 2, regulatory, cardiac, slow	Myl2	−10.85
1419145_at	smoothelin-like 1	Smtnl1	−9.68
1416713_at	tubulin polymerization-promoting protein family member 3	Tppp3	−4.56
1452766_at	tubulin polymerization promoting protein	Tppp	−3.91
1418395_at	solute carrier family 47, member 1	Slc47a1	−3.66
1418301_at	interferon regulatory factor 6	Irf6	−3.58
1418714_at	dual specificity phosphatase 8	Dusp8	−3.37
1418511_at	Dermatopontin	Dpt	−3.34
1455203_at	RIKEN cDNA A930003A15 gene	A930003A15Rik	−3.30
1417917_at	calponin 1	Cnn1	−3.25
Upregulated genes			
1438540_at	collagen, type XXV, alpha 1	Col25a1	6.51
1440085_at	ecto*dysp*lasin A2 receptor	Eda2r	5.73
1438059_at	cortexin 3	Ctxn3	5.23
1421698_a_at	collagen, type XIX, alpha 1	Col19a1	5.13
1456953_at	collagen, type XIX, alpha 1	Col19a1	5.11
1451203_at	myoglobin	Mb	4.75
1447807_s_at	pleckstrin homology domain containing, family H (with MyTH4 domain) member 1	Plekhh1	4.52
1422864_at	runt related transcription factor 1	Runx1	4.08
1422865_at	runt related transcription factor 1	Runx1	3.94
1418203_at	phorbol-12-myristate-13-acetate-induced protein 1	Pmaip1	3.69

**Table 2:** DEGs associated with signaling.

Probe Set ID	Gene Title	Gene Symbol	FC
MAPK signaling pathway			
1418714_at	dual specificity phosphatase 8	Dusp8	−3.37
1438933_x_at	RAS, guanyl releasing protein 2	Rasgrp2	−2.94
1419625_at	heat shock protein 1-like	Hspa1l	−2.81
1423100_at	FBJ osteosarcoma oncogene	Fos	−2.43
1448694_at	Jun oncogene	Jun	−2.07
1417164_at	dual specificity phosphatase 10	Dusp10	−2.06
1438883_at	fibroblast growth factor 5	Fgf5	−2.05
1427582_at	fibroblast growth factor 6	Fgf6	−2.03
1448830_at	dual specificity phosphatase 1	Dusp1	−1.95
1418401_a_at	dual specificity phosphatase 16	Dusp16	−1.76
1449117_at	Jun proto-oncogene related gene D	Jund	−1.75
1439205_at	nuclear factor of activated T cells, cytoplasmic, calcineurin dependent 2	Nfatc2	−1.64
1438030_at	RAS, guanyl releasing protein 3	Rasgrp3	−1.63
1449773_s_at	growth arrest and DNA-damage-inducible 45 beta	Gadd45b	−1.62
1435196_at	neurotrophic tyrosine kinase, receptor, type 2	Ntrk2	1.52
1421897_at	ELK1, member of ETS oncogene family	Elk1	1.56
1417856_at	avian reticuloendotheliosis viral (v-rel) oncogene related B	Relb	1.58
1421324_a_at	thymoma viral proto-oncogene 2	Akt2	1.65
1420895_at	transforming growth factor, beta receptor I	Tgfbr1	1.72
1440343_at	ribosomal protein S6 kinase, polypeptide 5	Rps6ka5	1.75
1436912_at	calcium channel, voltage-dependent, beta 4 subunit	Cacnb4	1.83
Wnt signaling pathway			
1449425_at	wingless-related MMTV integration site 2	Wnt2	−2.54
1423760_at	CD44 antigen	Cd44	−2.29
1451031_at	secreted frizzled-related protein 4	Sfrp4	−2.20
1418136_at	transforming growth factor beta 1 induced transcript 1	Tgfb1i1	−1.82
1427138_at	coiled-coil domain containing 88C	Ccdc88c	−1.80
1417985_at	Notch-regulated ankyrin repeat protein	Nrarp	−1.72
1455689_at	frizzled homolog 10 (Drosophila)	Fzd10	−1.56
1429506_at	naked cuticle 1 homolog (Drosophila)	Nkd1	1.53
1451689_a_at	SRY-box containing gene 10	Sox10	1.59
1460187_at	secreted frizzled-related protein 1	Sfrp1	2.36
PI3K and mTor signaling pathway			
1451038_at	apelin	Apln	−1.96
1449022_at	nestin	Nes	−1.62
1421679_a_at	cyclin-dependent kinase inhibitor 1A (P21)	Cdkn1a	1.52
1421324_a_at	thymoma viral proto-oncogene 2	Akt2	1.65
1425515_at	phosphatidylinositol 3-kinase, regulatory subunit, polypeptide 1 (p85 alpha)	Pik3r1	1.73
G protein coupled signaling			
1444409_at	rabphilin 3A-like (without C2 domains)	Rph3al	−2.37
1417625_s_at	chemokine (C-X-C motif) receptor 7	Cxcr7	−2.18
1455466_at	G protein-coupled receptor 133	Gpr133	−2.10
1451038_at	apelin	Apln	−1.96
1440009_at	olfactory receptor 78	Olfr78	−1.81
1431326_a_at	tropomodulin 2	Tmod2	−1.68
1455689_at	frizzled homolog 10 (Drosophila)	Fzd10	−1.56
1418394_a_at	CD97 antigen	Cd97	−1.56
1420940_x_at	regulator of G-protein signaling 5	Rgs5	−1.54
1417327_at	caveolin 2	Cav2	−1.53
1416286_at	regulator of G-protein signaling 4	Rgs4	−1.50
1460440_at	latrophilin 3	Lphn3	1.62
1451411_at	G protein-coupled receptor, family C, group 5, member B	Gprc5b	1.63
1456833_at	G protein-coupled receptor 17	Gpr17	1.68
1442082_at	complement component 3a receptor 1	C3ar1	1.81
1436912_at	calcium channel, voltage-dependent, beta 4 subunit	Cacnb4	1.83
1420401_a_at	receptor (calcitonin) activity modifying protein 3	Ramp3	1.86
1454782_at	brain-specific angiogenesis inhibitor 3	Bai3	2.02
1434172_at	cannabinoid receptor 1 (brain)	Cnr1	2.11
1432466_a_at	apolipoprotein E	Apoe	2.17
1460123_at	G protein-coupled receptor 1	Gpr1	2.37
1450875_at	G protein-coupled receptor 37	Gpr37	2.54
1436889_at	gamma-aminobutyric acid (GABA) A receptor, subunit alpha 1	Gabra1	2.54
Other transcription factors and transcriptional modulators			
1455267_at	estrogen-related receptor gamma	Esrrg	−3.04
1449363_at	activating transcription factor 3	Atf3	−2.58
1418572_x_at	tumor necrosis factor receptor superfamily, member 12a	Tnfrsf12a	−2.39
1418762_at	CD55 antigen	Cd55	−2.14
1425518_at	Rap guanine nucleotide exchange factor (GEF) 4	Rapgef4	−1.73
1422742_at	human immunodeficiency virus type I enhancer binding protein 1	Hivep1	−1.72
1420696_at	sema domain, immunoglobulin domain (Ig), short basic domain, secreted, (semaphorin) 3C	Sema3c	−1.68
1456796_at	snail homolog 3 (Drosophila)	Snai3	−1.66
1418936_at	v-maf musculoaponeurotic fibrosarcoma oncogene family, protein F (avian)	Maff	−1.61
1451932_a_at	ADAMTS-like 4	Adamtsl4	−1.58
1425896_a_at	fibrillin 1	Fbn1	−1.57
1418394_a_at	CD97 antigen	Cd97	−1.56
1459372_at	neuronal PAS domain protein 4	Npas4	−1.51
1424880_at	tribbles homolog 1 (Drosophila)	Trib1	−1.50
1428983_at	scleraxis	Scx	1.53
1429841_at	multiple EGF-like-domains 10	Megf10	1.53
1422210_at	forkhead box D3	Foxd3	1.57
1441107_at	doublesex and mab-3 related transcription factor like family A2	Dmrta2	1.58
1435775_at	circadian locomotor output cycles kaput	Clock	1.60
1457342_at	IKAROS family zinc finger 4	Ikzf4	1.60
1452650_at	tripartite motif-containing 62	Trim62	1.61
1449164_at	CD68 antigen	Cd68	1.61
1452021_a_at	hairy and enhancer of split 6	Hes6	1.66
1434458_at	follistatin	Fst	1.93
1450042_at	aristaless related homeobox	Arx	2.18
1454693_at	histone deacetylase 4	Hdac4	2.36
1418937_at	deiodinase, iodothyronine, type II	Dio2	2.87
1422864_at	runt related transcription factor 1	Runx1	4.08
1440085_at	ecto*dysp*lasin A2 receptor	Eda2r	5.73

**Table 3:** Muscle organ-related DEGs associated with muscle contraction and mechanical force production.

Muscle organ related differentially regulated in *dysp*				
(a) Muscle contraction/ mechanical force				
Probe Set ID	Gene Title	Gene Symbol	Fold Change	Localization/Function
1448394_at	myosin, light polypeptide 2, regulatory, cardiac, slow	Myl2	−10.85	Sarcomere, part of myosin filaments
1419145_at	smoothelin-like 1	Smtnl1	−9.68	Sarcomere, binds calmodulin and tropomyosin ^S1^
1417917_at	calponin 1	Cnn1	−3.25	Sarcomere; binds tropomyosin and inhibits cross-bridge cycle in a Ca2+ dependent manner ^S2^
1449996_a_at	tropomyosin 3, gamma	Tpm3	−3.24	Sarcomere, Actin filament associated
1420991_at	ankyrin repeat domain 1 (cardiac muscle)	Ankrd1	−2.99	Sarcomere, Z-disc, Part of titin-N2A mechanosensory complex ^S3^
1427768_s_at	myosin, light polypeptide 3	Myl3	−2.93	Sarcomere, part of myosin filaments
1439204_at	sodium channel, voltage-gated, type III, alpha	Scn3a	−2.93	Sarcolemma, Sodium channel
1452670_at	myosin, light polypeptide 9, regulatory	Myl9	−2.65	Sarcomere, part of myosin filaments
1420647_a_at	keratin 8	Krt8	−2.58	Sacomere; Z-disc and M-line domains at costameres at the sarcolemmal membrane ^S4^
1421253_at	nebulin-related anchoring protein	Nrap	−2.41	Sarcomere; Z-disc; terminal actin binding
1460318_at	cysteine and glycine-rich protein 3	Csrp3	−2.37	Sarcomere; Z-disc
1416554_at	PDZ and LIM domain 1 (elfin)	Pdlim1	−2.31	Sarcomere; Z-disc; Interaction with α-actinin
1435767_at	sodium channel, voltage-gated, type III, beta	Scn3b	−2.23	Sarcolemma, Sodium channel
1417872_at	four and a half LIM domains 1	Fhl1	−1.94	Sarcomere, Z-disc ^S5^
1416326_at	cysteine-rich protein 1 (intestinal)	Crip1	−1.76	Sarcomere, Z-disc; Interaction with α-actinin ^S5^
1422635_at	acetylcholinesterase	Ache	−1.71	
1450650_at	myosin X	Myo10	1.57	Link between integrins and cytoskeleton ^S6^
1424967_x_at	troponin T2, cardiac	Tnnt2	1.59	Sarcomere; interaction with tropomyosin of actin filaments
1449307_at	dysbindin (dystrobrevin binding protein 1) domain containing 1	Dbndd1	1.75	costameres, part of dystrophin-glycoprotein complex (DGC) ^S7^
1436912_at	calcium channel, voltage-dependent, beta 4 subunit	Cacnb4	1.83	neuronal Calcium channel subunit; able to associate with Ca_v_1.1 of skeletal muscle
1418852_at	cholinergic receptor, nicotinic, alpha polypeptide 1 (muscle)	Chrna1	2.28	Neuromuscular junctions; muscle excitation
(b) Structure and Morphogenesis				
Probe Set ID	Gene Title	Gene Symbol	Fold Change	Localization/Function
1418511_at	dermatopontin	Dpt	−3.34	cell-matrix adhesion ^S8^
1449082_at	microfibrillar associated protein 5	Mfap5	−3.13	ECM; glycoprotein associated with microbibrils like elastine ^S9^
1450798_at	tenascin XB	Tnxb	−2.85	ECM; collagen formation ^S10^
1456344_at	tenascin C	Tnc	−2.63	ECM; glycoprotein; interaction with fibronectin ^S11^
1416697_at	dipeptidylpeptidase 4	Dpp4	−2.25	cell surface peptidase; cell-cell connections ^S12^
1424701_at	protocadherin 20	Pcdh20	−2.35	Transmembrane protein, cell-cell connentions
1423760_at	CD44 antigen	Cd44	−2.29	Cell surface glycoprotein; migration and myoblast fusion ^S13^
1449388_at	thrombospondin 4	Thbs4	−2.14	ECM glycoprotein
1426529_a_at	transgelin 2	Tagln2	−1.93	Cytoskeleton; Actin-gelling protein ^S14^
1437218_at	fibronectin 1	Fn1	−1.74	ECM glycoprotein, cell adhesion
1434928_at	growth arrest-specific 2 like 1	Gas2l1	−1.72	Cytoskeletaon; Crosslinking of microfilaments and microtubules 15
1449022_at	nestin	Nes	−1.62	Cytoskeleton, intermediate filament, colocalized with desmin in Z-disc of embryonic skeletal muscle ^S16^
1451932_a_at	ADAMTS-like 4	Adamtsl4	−1.58	ECM; glycoprotein; microfibril biogenesis ^S17^
1425896_a_at	fibrillin 1	Fbn1	−1.57	ECM glycoprotein
1436425_at	KN motif and ankyrin repeat domains 4	Kank4	1.56	Control of actin-polymerization ^S18^
1434709_at	neuron-glia-CAM-related cell adhesion molecule	Nrcam	1.64	Transmembrane cell adhesion protein; axon guidance ^S19^
1418204_s_at	allograft inflammatory factor 1	Aif1	1.68	Actin-polymerizing protein ^S20^
1419050_at	transmembrane protein 8C	Tmem8c	1.74	Transmembrane cell surface protein, myoblast fusion ^S21^
1429861_at	protocadherin 9	Pcdh9	1.90	Transmembrane protein, cell-cell connentions
1418139_at	doublecortin	Dcx	2.03	Marker for Pax7+MyoD− subpopulation contributing to myofiber maturation during muscle regeneration ^S22^
1456953_at	collagen, type XIX, alpha 1	Col19a1	5.11	ECM, expressed during muscle development ^S23^
1438540_at	collagen, type XXV, alpha 1	Col25a1	6.51	ECM, branching of axon bundles within the muscle ^S24^

(**a**), and muscle structure/morphogenesis (**b**). Corresponding references are given in Supplementary Table S3.

**Table 4:** Primer sequences and amplicon size used for qRT-PCR.

Gene	Primers (5′ to 3′)	Amplicon (bp)
*Bai3*	Fwd: AGTATGGAGGAAGGCCCTGT	107
	Rev: GTGGCTCCATGAACTCCATT	
*Col19a1*	Fwd: TTGGATTGCCAGGAGAACAT	114
	Rev: CAGCATCACCCTTCAGACCT	
*Flcn*	Fwd: GCTGGGATTACCGAACTGAG	110
	Rev: AGGCGATCTGTCGTAACACC	
*Fos*	Fwd: AGTCAAGGCCTGGTCTGTGT	100
	Rev: TCCAGCACCAGGTTAATTCC	
*Gapdh*	Fwd: AGTGTTTCCTCGTCCCGTAG	119
	Rev: TGATGGCAACAATCTCCACT	
*Jun*	Fwd: GAAAAGTAGCCCCCAACCTC	106
	Rev: ACAGGGGACACAGCTTTCAC	
*Mrf4*	Fwd: GCAGAGGGCTCTCCTTTGTA	105
	Rev: AACGTGTTCCTCTCCACTGC	
*Myf5*	Fwd: GAAGGTCAACCAAGCTTTCG	109
	Rev: GCTCTCAATGTAGCGGATGG	
*Myl2*	Fwd: AAAGAGGCTCCAGGTCCAAT	105
	Rev: CACCTTGAATGCGTTGAGAA	
*Myod*	Fwd: GGCTACGACACCGCCTACTA	110
	Rev: GTGGAGATGCGCTCCACTAT	
*Myog*	Fwd: CTGCACTCCCTTACGTCCAT	103
	Rev: CCCAGCCTGACAGACAATCT	
*Pax3*	Fwd: AAACCCAAGCAGGTGACAAC	115
	Rev: AGACAGCGTCCTTGAGCAAT	
*Pax7*	Fwd: ATTACCTGGCCAAAAACGTG	105
	Rev: AGTAGGCTTGTCCCGTTTCC	
*Six1*	Fwd: CCTGGGGCAAAATGATGTAT	112
	Rev: CAAAGCATGAGCAAGCCAAC	
*Six4*	Fwd: GGCCAGAGGTTGTTGTTTGT	109
	Rev: GGCAGCCAAGCTGTGTAAGT	
*Trib1*	Fwd: TAACAAACTCCCCCTTGCTG	105
	Rev: CAACGCAGAACAGTCATGGT	
