# Corrigendum: Gene profiling of embryonic skeletal muscle lacking type I ryanodine receptor Ca^2+^ release channel

**DOI:** 10.1038/srep24450

**Published:** 2016-04-22

**Authors:** Dilyana Filipova, Anna M. Walter, John A. Gaspar, Anna Brunn, Nina F. Linde, Mostafa A. Ardestani, Martina Deckert, Jürgen Hescheler, Gabriele Pfitzer, Agapios Sachinidis, Symeon Papadopoulos

Scientific Reports 6: Article number: 2005010.1038/srep20050; published online: 02012016; updated: 04222016

This Article contains a typographical error in the Methods section under subheading ‘Animals and skeletal muscle preparation’.

“All experimental protocols were approved by the local governmental authorities (Landesamt für Natur, Umwelt und Verbraucherschutz, North Rhine-Westphalia, AZ84-02.05.20.13.010).”

should read:

“All experimental protocols were approved by the local governmental authorities (Landesamt für Natur, Umwelt und Verbraucherschutz, North Rhine-Westphalia, AZ84-02.05.20.13.080).”

