# Supplementary material for: Gene profiling of embryonic skeletal muscle lacking type I ryanodine receptor Ca2+ release channel
Source: Sci Rep. 2016 Feb 1;6:20050. doi: 10.1038/srep20050 (PMC4735524; doi:10.1038/srep20050)
Supplement: Supplementary Information [file srep20050-s1.pdf]

Supplementary information to:
Gene profiling of embryonic skeletal muscle lacking type I ryanodine receptor Ca^{2+} release channel

Dilyana Filipova, Anna M. Walter, John A. Gaspar, Anna Brunn, Nina F. Linde, Mostafa A. Ardestani, Martina Deckert, Jürgen Hescheler, Gabriele Pfitzer, Agapios Sachinidis, Symeon Papadopoulos

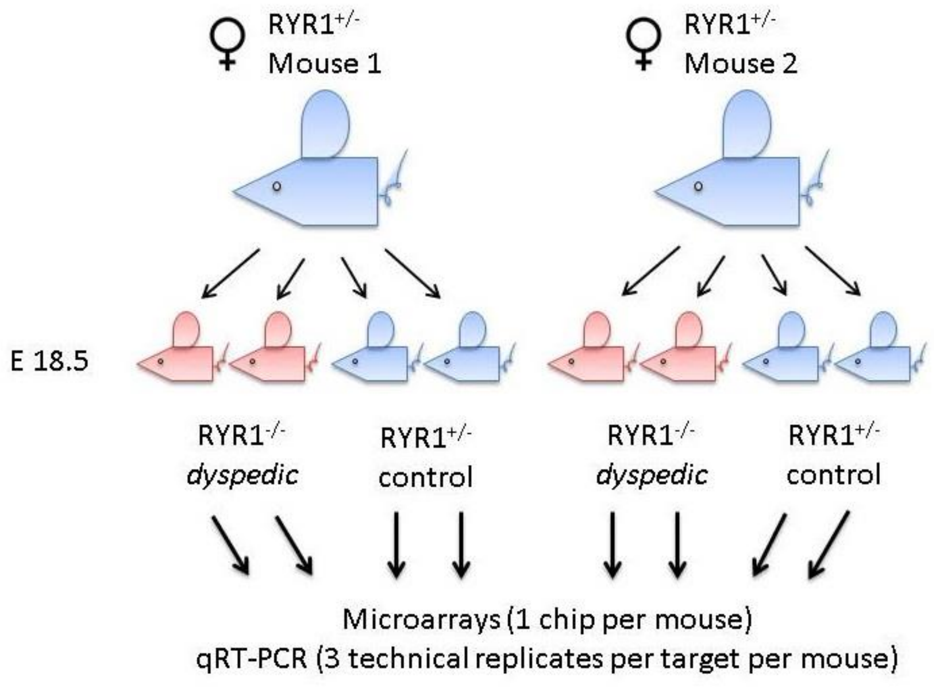

Supplementary Figure S1: A graphical representation of the biological replicates used for the microarray and qRT-PCR analyses

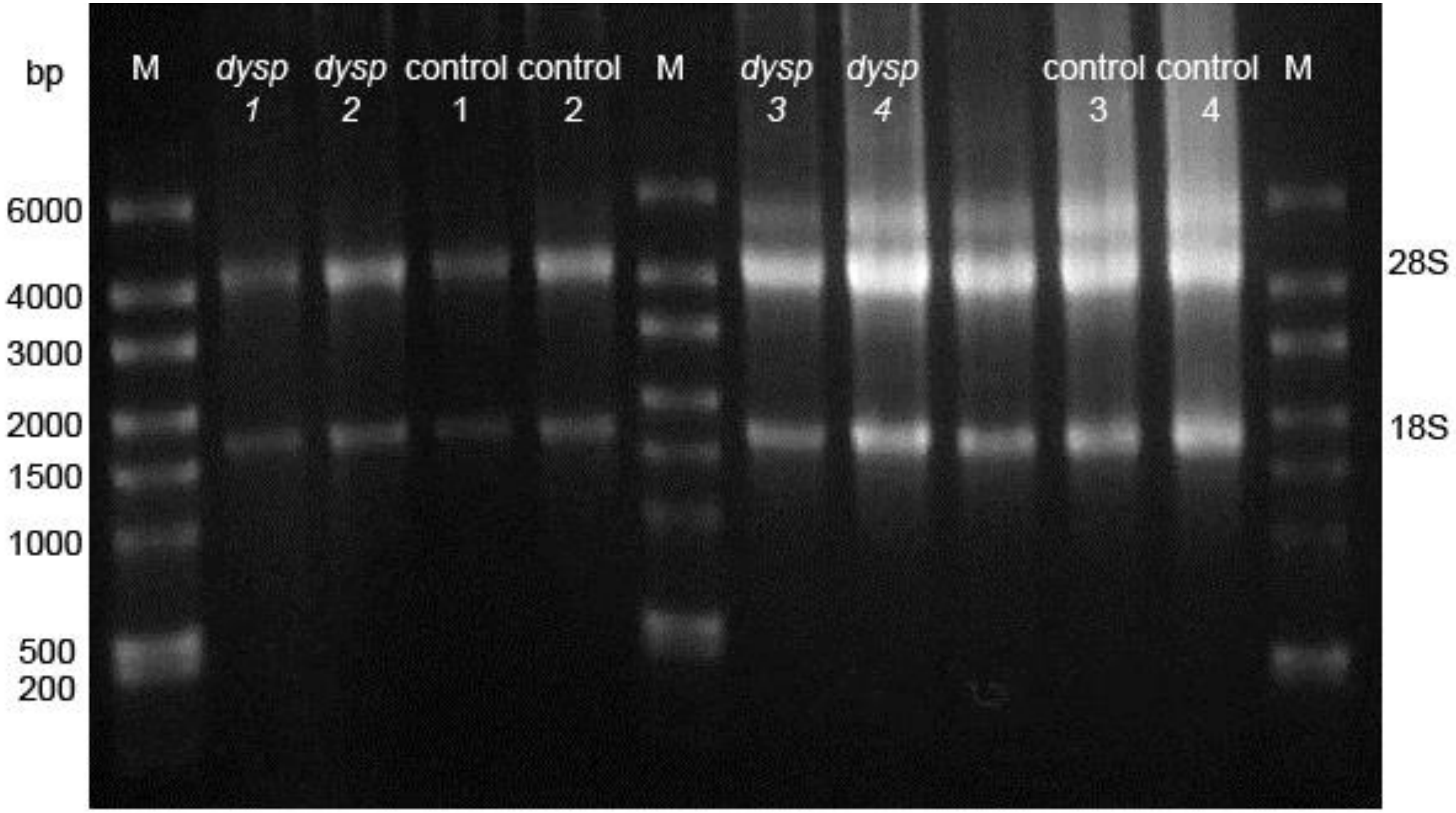

Sample	c [ng/µl]	Sample	c [ng/µl]
dysp 1	142.3	dysp 3	458.6
dysp 2	224.0	dysp 4	434.0
control 1	183.9	control 3	835.2
control 2	278.3	control 4	934.7

Supplementary Figure S2: 5 µl aliquots of the extracted RNAs (*dysp* 1-4 and control 1-4) were applied on a 2 % agarose gel and subjected to an electrophoresis at 100 mV for 90 minutes next to 5 µl RiboRuler High Range RNA Ladder (marked as "M") (Thermo Fisher Scientific). Distinct bands corresponding to the 28S and 18S RNAs were observed. The concentrations of the RNA samples are given in the table.

Supplementary Table S1: Genes, identified as differentially regulated in the microarray analysis, displaying a FC greater than ± 1.5 and adjusted P value ≤ 0.05

Probe Set ID	Gene Title	Gene Symbol	Fold Change Value (Homozygote vs Heterozygote)	t-Score	P.Value	adj.P.Val
**1448394_at**	myosin, light polypeptide 2, regulatory, cardiac, slow	Myl2	-10.85	-23.44	0.00	0.00
**1419145_at**	smoothelin-like 1	Smtnl1	-9.68	-12.10	0.00	0.00
**1416713_at**	tubulin polymerization-promoting protein family member 3	Tppp3	-4.56	-18.68	0.00	0.00
**1452766_at**	tubulin polymerization promoting protein	Tppp	-3.91	-15.96	0.00	0.00
**1418395_at**	solute carrier family 47, member 1	Slc47a1	-3.66	-7.79	0.00	0.01
**1418301_at**	interferon regulatory factor 6	Irf6	-3.58	-9.99	0.00	0.00
**1418714_at**	dual specificity phosphatase 8	Dusp8	-3.37	-8.60	0.00	0.01
**1418511_at**	dermatopontin	Dpt	-3.34	-11.34	0.00	0.00
**1455203_at**	RIKEN cDNA A930003A15 gene	A930003A15Rik	-3.30	-8.85	0.00	0.00
**1417917_at**	calponin 1	Cnn1	-3.25	-12.18	0.00	0.00
**1449996_a_at**	tropomyosin 3, gamma	Tpm3	-3.24	-10.08	0.00	0.00
**1429598_at**	RIKEN cDNA 2310042D19 gene	2310042D19Rik	-3.22	-6.66	0.00	0.01
**1449997_at**	tropomyosin 3, gamma	Tpm3	-3.22	-10.60	0.00	0.00
**1441636_at**	---	---	-3.17	-16.45	0.00	0.00
**1423285_at**	coagulation factor C homolog (Limulus polyphemus)	Coch	-3.17	-8.80	0.00	0.00
**1449082_at**	microfibrillar associated protein 5	Mfap5	-3.13	-19.37	0.00	0.00
**1433529_at**	peptidase domain containing associated with muscle regeneration 1	Pamr1	-3.09	-7.59	0.00	0.01
**1455267_at**	estrogen-related receptor gamma	Esrrg	-3.04	-8.19	0.00	0.01

**1420991_at**	ankyrin repeat domain 1 (cardiac muscle)	Ankrd1	-2.99	-5.98	0.00	0.02
**1438933_x_at**	RAS, guanyl releasing protein 2	Rasgrp2	-2.94	-13.58	0.00	0.00
**1427768_s_at**	myosin, light polypeptide 3	Myl3	-2.93	-5.55	0.00	0.03
**1439204_at**	sodium channel, voltage-gated, type III, alpha	Scn3a	-2.93	-5.86	0.00	0.03
**1450798_at**	tenascin XB	Tnxb	-2.85	-7.43	0.00	0.01
**1428266_at**	myosin, light polypeptide 3	Myl3	-2.84	-6.01	0.00	0.02
**1419625_at**	heat shock protein 1-like	Hspa1l	-2.81	-9.70	0.00	0.00
**1418454_at**	microfibrillar associated protein 5	Mfap5	-2.80	-15.02	0.00	0.00
**1440527_at**	---	---	-2.80	-6.95	0.00	0.01
**1428861_at**	filamin A interacting protein 1-like	Filip1l	-2.78	-12.11	0.00	0.00
**1426851_a_at**	nephroblastoma overexpressed gene	Nov	-2.67	-11.01	0.00	0.00
**1454613_at**	dihydropyrimidinase-like 3	Dpysl3	-2.66	-10.17	0.00	0.00
**1452670_at**	myosin, light polypeptide 9, regulatory	Myl9	-2.65	-15.91	0.00	0.00
**1426852_x_at**	nephroblastoma overexpressed gene	Nov	-2.63	-10.40	0.00	0.00
**1456344_at**	tenascin C	Tnc	-2.63	-6.99	0.00	0.01
**1428960_at**	enkurin, TRPC channel interacting protein	Enkur	-2.61	-7.82	0.00	0.01
**1446380_at**	RIKEN cDNA 9430076C15 gene	9430076C15Rik	-2.59	-6.08	0.00	0.02
**1434094_at**	transmembrane protein 125	Tmem125	-2.59	-5.51	0.00	0.03
**1420647_a_at**	keratin 8	Krt8	-2.58	-10.75	0.00	0.00
**1449363_at**	activating transcription factor 3	Atf3	-2.58	-5.18	0.00	0.04
**1423691_x_at**	keratin 8	Krt8	-2.56	-9.40	0.00	0.00
**1449425_at**	wingless-related MMTV integration site 2	Wnt2	-2.54	-5.87	0.00	0.03
**1427769_x_at**	myosin, light polypeptide 3	Myl3	-2.53	-6.00	0.00	0.02
**1416342_at**	tenascin C	Tnc	-2.51	-5.93	0.00	0.02
**1423537_at**	growth associated protein 43	Gap43	-2.45	-8.11	0.00	0.01
**1425848_a_at**	dual specificity phosphatase 26 (putative)	Dusp26	-2.43	-8.08	0.00	0.01
**1423100_at**	FBJ osteosarcoma oncogene	Fos	-2.43	-6.47	0.00	0.02
**1452244_at**	mesenteric estrogen dependent adipogenesis	Medag	-2.43	-9.31	0.00	0.00
**1418591_at**	DnaJ (Hsp40) homolog, subfamily A, member 4	Dnaja4	-2.42	-9.56	0.00	0.00

**1421253_at**	nebulin-related anchoring protein	Nrap	-2.41	-4.96	0.00	0.05
**1426937_at**	mesenteric estrogen dependent adipogenesis	Medag	-2.39	-5.56	0.00	0.03
**1418572_x_at**	tumor necrosis factor receptor superfamily, member 12a	Tnfrsf12a	-2.39	-5.16	0.00	0.04
**1435571_at**	myelin protein zero-like 3	Mpzl3	-2.37	-6.77	0.00	0.01
**1460318_at**	cysteine and glycine-rich protein 3	Csrp3	-2.37	-8.41	0.00	0.01
**1444409_at**	rabphilin 3A-like (without C2 domains)	Rph3al	-2.37	-6.08	0.00	0.02
**1424701_at**	protocadherin 20	Pcdh20	-2.35	-10.43	0.00	0.00
**1422927_at**	Yip1 domain family, member 7	Yipf7	-2.33	-5.78	0.00	0.03
**1416554_at**	PDZ and LIM domain 1 (elfin)	Pdlim1	-2.31	-6.74	0.00	0.01
**1423760_at**	CD44 antigen	Cd44	-2.29	-11.65	0.00	0.00
**1451418_a_at**	splA/ryanodine receptor domain and SOCS box containing 4	Spsb4	-2.29	-9.26	0.00	0.00
**1415877_at**	dihydropyrimidinase-like 3	Dpysl3	-2.27	-9.51	0.00	0.00
**1430194_at**	pentraxin 4	Ptx4	-2.26	-6.27	0.00	0.02
**1416697_at**	dipeptidylpeptidase 4	Dpp4	-2.25	-5.17	0.00	0.04
**1438932_at**	RAS, guanyl releasing protein 2	Rasgrp2	-2.25	-6.22	0.00	0.02
**1438789_s_at**	dihydropyrimidinase-like 3	Dpysl3	-2.24	-12.16	0.00	0.00
**1435767_at**	sodium channel, voltage-gated, type III, beta	Scn3b	-2.23	-8.13	0.00	0.01
**1418923_at**	solute carrier family 17 (sodium phosphate), member 3	Slc17a3	-2.22	-7.09	0.00	0.01
**1453839_a_at**	peptidase inhibitor 16	Pi16	-2.22	-6.81	0.00	0.01
**1434376_at**	CD44 antigen	Cd44	-2.22	-11.86	0.00	0.00
**1435989_x_at**	keratin 8	Krt8	-2.20	-6.89	0.00	0.01
**1427975_at**	RAS-like, family 10, member A	Rasl10a	-2.20	-6.86	0.00	0.01
**1451031_at**	secreted frizzled-related protein 4	Sfrp4	-2.20	-6.08	0.00	0.02
**1417625_s_at**	chemokine (C-X-C motif) receptor 7	Cxcr7	-2.18	-4.98	0.00	0.05
**1418105_at**	stathmin-like 4	Stmn4	-2.18	-8.39	0.00	0.01
**1416069_at**	phosphofructokinase, platelet	Pfkp	-2.17	-5.46	0.00	0.03

**1434196_at**	DnaJ (Hsp40) homolog, subfamily A, member 4	Dnaja4	-2.17	-6.05	0.00	0.02
**1449280_at**	endothelial cell-specific molecule 1	Esm1	-2.16	-6.88	0.00	0.01
**1419647_a_at**	immediate early response 3	Ier3	-2.14	-5.60	0.00	0.03
**1449388_at**	thrombospondin 4	Thbs4	-2.14	-7.17	0.00	0.01
**1418762_at**	CD55 antigen	Cd55	-2.14	-9.16	0.00	0.00
**1460684_at**	transmembrane 7 superfamily member 2	Tm7sf2	-2.14	-8.96	0.00	0.00
**1417933_at**	insulin-like growth factor binding protein 6	Igfbp6	-2.10	-7.08	0.00	0.01
**1455466_at**	G protein-coupled receptor 133	Gpr133	-2.10	-7.41	0.00	0.01
**1451419_at**	splA/ryanodine receptor domain and SOCS box containing 4	Spsb4	-2.07	-7.00	0.00	0.01
**1448694_at**	Jun oncogene	Jun	-2.07	-7.47	0.00	0.01
**1426328_a_at**	sodium channel, voltage-gated, type III, beta	Scn3b	-2.07	-7.23	0.00	0.01
**1417164_at**	dual specificity phosphatase 10	Dusp10	-2.06	-13.52	0.00	0.00
**1424567_at**	tetraspanin 2	Tspan2	-2.06	-7.47	0.00	0.01
**1438883_at**	fibroblast growth factor 5	Fgf5	-2.05	-5.86	0.00	0.03
**1427582_at**	fibroblast growth factor 6	Fgf6	-2.03	-7.73	0.00	0.01
**1432509_at**	RIKEN cDNA 5033430I15 gene	5033430I15Rik	-2.02	-5.99	0.00	0.02
**1453321_at**	fibronectin type III domain containing 1	Fndc1	-2.01	-9.58	0.00	0.00
**1417860_a_at**	spondin 2, extracellular matrix protein	Spon2	-1.99	-7.21	0.00	0.01
**1422760_at**	peptidyl arginine deiminase, type IV	Padi4	-1.98	-7.50	0.00	0.01
**1451038_at**	apelin	Apln	-1.96	-7.62	0.00	0.01
**1418424_at**	tumor necrosis factor alpha induced protein 6	Tnfaip6	-1.96	-5.37	0.00	0.04
**1448830_at**	dual specificity phosphatase 1	Dusp1	-1.95	-12.24	0.00	0.00
**1418270_at**	a disintegrin-like and metallopeptidase (reprolysin type) with thrombospondin type 1 motif, 8	Adamts8	-1.95	-7.82	0.00	0.01
**1417872_at**	four and a half LIM domains 1	Fhl1	-1.94	-9.74	0.00	0.00
**1417673_at**	growth factor receptor bound protein 14	Grb14	-1.93	-5.93	0.00	0.02
**1426529_a_at**	transgelin 2	Tagln2	-1.93	-11.63	0.00	0.00

**1448779_at**	CDKN1A interacting zinc finger protein 1	Ciz1	-1.92	-6.28	0.00	0.02
**1447822_x_at**	UFM1-specific peptidase 1	Ufsp1	-1.91	-4.96	0.00	0.05
**1418954_at**	calcium/calmodulin-dependent protein kinase kinase 1, alpha	Camkk1	-1.90	-8.72	0.00	0.00
**1417409_at**	Jun oncogene	Jun	-1.88	-6.23	0.00	0.02
**1423180_at**	potassium voltage gated channel, Shab-related subfamily, member 1	Kcnb1	-1.87	-5.64	0.00	0.03
**1424568_at**	tetraspanin 2	Tspan2	-1.87	-11.69	0.00	0.00
**1453128_at**	lymphatic vessel endothelial hyaluronan receptor 1	Lyve1	-1.86	-5.31	0.00	0.04
**1445454_at**	DNA segment, Chr 2, ERATO Doi 282, expressed	D2Ertd282e	-1.86	-5.89	0.00	0.02
**1415806_at**	plasminogen activator, tissue	Plat	-1.84	-5.53	0.00	0.03
**1435396_at**	syntaxin binding protein 6 (amisyn)	Stxbp6	-1.84	-11.13	0.00	0.00
**1436293_x_at**	immunoglobulin-like domain containing receptor 2	Ildr2	-1.83	-9.11	0.00	0.00
**1418136_at**	transforming growth factor beta 1 induced transcript 1	Tgfb1i1	-1.82	-9.21	0.00	0.00
**1435378_at**	small integral membrane protein 5	Smim5	-1.81	-6.88	0.00	0.01
**1460330_at**	annexin A3	Anxa3	-1.81	-7.78	0.00	0.01
**1440009_at**	olfactory receptor 78	Olfr78	-1.81	-6.21	0.00	0.02
**1429379_at**	lymphatic vessel endothelial hyaluronan receptor 1	Lyve1	-1.81	-6.32	0.00	0.02
**1427138_at**	coiled-coil domain containing 88C	Ccdc88c	-1.80	-7.97	0.00	0.01
**1452483_a_at**	CD44 antigen	Cd44	-1.80	-5.10	0.00	0.04
**1452803_at**	GLI pathogenesis-related 2	Glipr2	-1.80	-6.62	0.00	0.02
**1448147_at**	tumor necrosis factor receptor superfamily, member 19	Tnfrsf19	-1.79	-5.20	0.00	0.04
**1441338_at**	RIKEN cDNA 5930412G12 gene	5930412G12Rik	-1.78	-6.21	0.00	0.02
**1417807_at**	UFM1-specific peptidase 1	Ufsp1	-1.78	-5.94	0.00	0.02
**1426784_at**	tripartite motif-containing 47	Trim47	-1.78	-8.88	0.00	0.00

**1452067_at**	N-acylethanolamine acid amidase	Naaa	-1.77	-5.64	0.00	0.03
**1442862_at**	---	---	-1.76	-5.83	0.00	0.03
**1416326_at**	cysteine-rich protein 1 (intestinal)	Crip1	-1.76	-9.74	0.00	0.00
**1418401_a_at**	dual specificity phosphatase 16	Dusp16	-1.76	-9.35	0.00	0.00
**1454997_at**	methionine sulfoxide reductase B3	Msrb3	-1.76	-8.21	0.00	0.01
**1449117_at**	Jun proto-oncogene related gene d	Jund	-1.75	-10.69	0.00	0.00
**1444174_at**	predicted gene 10700	Gm10700	-1.75	-6.12	0.00	0.02
**1455224_at**	angiopoietin-like 1	Angptl1	-1.74	-5.61	0.00	0.03
**1437218_at**	fibronectin 1	Fn1	-1.74	-6.45	0.00	0.02
**1451177_at**	DnaJ (Hsp40) homolog, subfamily B, member 4	Dnajb4	-1.73	-5.41	0.00	0.03
**1425518_at**	Rap guanine nucleotide exchange factor (GEF) 4	Rapgef4	-1.73	-5.04	0.00	0.05
**1439407_x_at**	transgelin 2	Tagln2	-1.73	-8.73	0.00	0.00
**1417985_at**	Notch-regulated ankyrin repeat protein	Nrarp	-1.72	-6.69	0.00	0.01
**1434928_at**	growth arrest-specific 2 like 1	Gas2l1	-1.72	-7.00	0.00	0.01
**1422742_at**	human immunodeficiency virus type I enhancer binding protein 1	Hivep1	-1.72	-7.48	0.00	0.01
**1434600_at**	tight junction protein 2	Tjp2	-1.72	-8.59	0.00	0.01
**1422635_at**	acetylcholinesterase	Ache	-1.71	-8.41	0.00	0.01
**1428864_at**	dual specificity phosphatase 8	Dusp8	-1.71	-5.22	0.00	0.04
**1425749_at**	syntaxin binding protein 6 (amisyn)	Stxbp6	-1.70	-6.46	0.00	0.02
**1426791_at**	RUN and SH3 domain containing 2	Rusc2	-1.68	-7.92	0.00	0.01
**1420696_at**	sema domain, immunoglobulin domain (Ig), short basic domain, secreted, (semaphorin) 3C	Sema3c	-1.68	-6.84	0.00	0.01
**1426964_at**	ABRA C-terminal like /// predicted pseudogene 6314	Abracl /// Gm6314	-1.68	-10.55	0.00	0.00
**1441228_at**	apolipoprotein L domain containing 1	Apold1	-1.68	-6.92	0.00	0.01
**1431326_a_at**	tropomodulin 2	Tmod2	-1.68	-5.03	0.00	0.05
**1417283_at**	Ly6/neurotoxin 1	Lynx1	-1.67	-6.79	0.00	0.01
**1434599_a_at**	tight junction protein 2	Tjp2	-1.67	-6.93	0.00	0.01

**1448927_at**	potassium intermediate/small conductance calcium-activated channel, subfamily N, member 2	Kcnn2	-1.67	-5.78	0.00	0.03
**1434188_at**	solute carrier family 16 (monocarboxylic acid transporters), member 12	Slc16a12	-1.66	-5.00	0.00	0.05
**1448289_at**	collapsin response mediator protein 1	Crmp1	-1.66	-5.90	0.00	0.02
**1440615_at**	dual specificity phosphatase 16	Dusp16	-1.66	-6.87	0.00	0.01
**1450612_a_at**	phosphatidylethanolamine N-methyltransferase	Pemt	-1.66	-6.03	0.00	0.02
**1456796_at**	snail homolog 3 (Drosophila)	Snai3	-1.66	-5.18	0.00	0.04
**1426938_at**	neuro-oncological ventral antigen 1	Nova1	-1.65	-7.33	0.00	0.01
**1419149_at**	serine (or cysteine) peptidase inhibitor, clade E, member 1	Serpine1	-1.65	-5.19	0.00	0.04
**1436212_at**	transmembrane protein 71	Tmem71	-1.65	-5.68	0.00	0.03
**1417889_at**	apolipoprotein B mRNA editing enzyme, catalytic polypeptide 2	Apobec2	-1.64	-6.87	0.00	0.01
**1439205_at**	nuclear factor of activated T cells, cytoplasmic, calcineurin dependent 2	Nfatc2	-1.64	-8.47	0.00	0.01
**1423321_at**	myeloid-associated differentiation marker	Myadm	-1.63	-5.95	0.00	0.02
**1429348_at**	sema domain, immunoglobulin domain (Ig), short basic domain, secreted, (semaphorin) 3C	Sema3c	-1.63	-7.62	0.00	0.01
**1436221_at**	immunoglobulin-like domain containing receptor 2	Ildr2	-1.63	-5.96	0.00	0.02
**1438030_at**	RAS, guanyl releasing protein 3	Rasgrp3	-1.63	-7.17	0.00	0.01
**1449773_s_at**	growth arrest and DNA-damage-inducible 45 beta	Gadd45b	-1.62	-5.32	0.00	0.04
**1441952_x_at**	Ly6/neurotoxin 1	Lynx1	-1.62	-7.93	0.00	0.01
**1439036_a_at**	ATPase, Na+/K+ transporting, beta 1 polypeptide	Atp1b1	-1.62	-8.06	0.00	0.01
**1449022_at**	nestin	Nes	-1.62	-7.35	0.00	0.01
**1444013_at**	RIKEN cDNA 5930412G12 gene	5930412G12Rik	-1.61	-6.37	0.00	0.02

**1418936_at**	v-maf musculoaponeurotic fibrosarcoma oncogene family, protein F (avian)	Maff	-1.61	-8.01	0.00	0.01
**1419703_at**	collagen, type V, alpha 3	Col5a3	-1.61	-5.90	0.00	0.02
**1426792_s_at**	RUN and SH3 domain containing 2	Rusc2	-1.60	-6.88	0.00	0.01
**1450985_a_at**	tight junction protein 2	Tjp2	-1.60	-6.20	0.00	0.02
**1423054_at**	WD repeat domain 1	Wdr1	-1.60	-6.42	0.00	0.02
**1446847_at**	coiled-coil domain containing 88C	Ccdc88c	-1.59	-7.12	0.00	0.01
**1431004_at**	lysyl oxidase-like 2	Loxl2	-1.58	-6.74	0.00	0.01
**1451932_a_at**	ADAMTS-like 4	Adamtsl4	-1.58	-4.98	0.00	0.05
**1425896_a_at**	fibrillin 1	Fbn1	-1.57	-5.45	0.00	0.03
**1417804_at**	RAS, guanyl releasing protein 2	Rasgrp2	-1.57	-6.03	0.00	0.02
**1455689_at**	frizzled homolog 10 (Drosophila)	Fzd10	-1.56	-6.94	0.00	0.01
**1418394_a_at**	CD97 antigen	Cd97	-1.56	-8.35	0.00	0.01
**1418289_at**	nestin	Nes	-1.55	-7.92	0.00	0.01
**1436194_at**	PRELI domain containing 2	Prelid2	-1.55	-5.12	0.00	0.04
**1435195_at**	vasohibin 1	Vash1	-1.55	-5.76	0.00	0.03
**1423890_x_at**	ATPase, Na+/K+ transporting, beta 1 polypeptide	Atp1b1	-1.55	-7.13	0.00	0.01
**1449016_at**	zona pellucida glycoprotein 2	Zp2	-1.54	-5.22	0.00	0.04
**1434092_at**	autophagy related 9B	Atg9b	-1.54	-5.10	0.00	0.04
**1438031_at**	RAS, guanyl releasing protein 3	Rasgrp3	-1.54	-4.95	0.00	0.05
**1420940_x_at**	regulator of G-protein signaling 5	Rgs5	-1.54	-5.14	0.00	0.04
**1417327_at**	caveolin 2	Cav2	-1.53	-6.15	0.00	0.02
**1427164_at**	interleukin 13 receptor, alpha 1	Il13ra1	-1.53	-5.19	0.00	0.04
**1450958_at**	transmembrane 4 superfamily member 1	Tm4sf1	-1.53	-5.29	0.00	0.04
**1451152_a_at**	ATPase, Na+/K+ transporting, beta 1 polypeptide	Atp1b1	-1.52	-6.85	0.00	0.01
**1450984_at**	tight junction protein 2	Tjp2	-1.52	-6.79	0.00	0.01
**1435123_at**	EFR3 homolog B (S. cerevisiae)	Efr3b	-1.52	-6.32	0.00	0.02

**1429239_a_at**	StAR-related lipid transfer (START) domain containing 4	Stard4	-1.52	-6.69	0.00	0.01
**1420679_a_at**	androgen-induced 1	Aig1	-1.52	-5.68	0.00	0.03
**1416480_a_at**	HIG1 domain family, member 1A	Higd1a	-1.52	-7.27	0.00	0.01
**1460208_at**	fibrillin 1	Fbn1	-1.51	-6.73	0.00	0.01
**1450851_at**	WD repeat domain 1	Wdr1	-1.51	-7.86	0.00	0.01
**1459372_at**	neuronal PAS domain protein 4	Npas4	-1.51	-7.37	0.00	0.01
**1418453_a_at**	ATPase, Na+/K+ transporting, beta 1 polypeptide	Atp1b1	-1.51	-6.91	0.00	0.01
**1428078_at**	ring finger protein, transmembrane 1	Rnft1	-1.51	-4.95	0.00	0.05
**1424880_at**	tribbles homolog 1 (Drosophila)	Trib1	-1.50	-5.67	0.00	0.03
**1453330_at**	coiled-coil domain containing 88C	Ccdc88c	-1.50	-6.13	0.00	0.02
**1416286_at**	regulator of G-protein signaling 4	Rgs4	-1.50	-7.45	0.00	0.01
**1452696_a_at**	RIKEN cDNA 4933439C10 gene	4933439C10Rik	1.50	6.85	0.00	0.01
**1459145_at**	RIKEN cDNA A930033H14 gene	A930033H14Rik	1.50	7.17	0.00	0.01
**1423663_at**	folliculin	Flcn	1.50	5.36	0.00	0.04
**1434809_at**	Rho GTPase activating protein 28	Arhgap28	1.51	6.75	0.00	0.01
**1420545_a_at**	chimerin (chimaerin) 1	Chn1	1.51	5.80	0.00	0.03
**1434542_at**	glutamic pyruvate transaminase (alanine aminotransferase) 2	Gpt2	1.51	6.93	0.00	0.01
**1425265_a_at**	exoribonuclease 3	Eri3	1.51	7.38	0.00	0.01
**1449331_a_at**	receptor-associated protein of the synapse	Rapsn	1.51	7.10	0.00	0.01
**1435196_at**	neurotrophic tyrosine kinase, receptor, type 2	Ntrk2	1.52	5.57	0.00	0.03
**1421679_a_at**	cyclin-dependent kinase inhibitor 1A (P21)	Cdkn1a	1.52	7.06	0.00	0.01
**1454880_s_at**	BCL2 modifying factor	Bmf	1.52	6.89	0.00	0.01
**1430313_at**	ADAMTS-like 1	Adamtsl1	1.53	5.91	0.00	0.02
**1445068_at**	mucosa associated lymphoid tissue lymphoma translocation gene 1	Malt1	1.53	9.54	0.00	0.00
**1429506_at**	naked cuticle 1 homolog (Drosophila)	Nkd1	1.53	6.66	0.00	0.01
**1428983_at**	scleraxis	Scx	1.53	5.51	0.00	0.03

**1451361_a_at**	patatin-like phospholipase domain containing 7	Pnpla7	1.53	6.18	0.00	0.02
**1439602_at**	fidgetin	Fign	1.53	5.86	0.00	0.03
**1424975_at**	sialic acid binding Ig-like lectin 5	Siglec5	1.53	6.27	0.00	0.02
**1429841_at**	multiple EGF-like-domains 10	Megf10	1.53	5.76	0.00	0.03
**1439327_at**	collagen and calcium binding EGF domains 1	Ccbe1	1.54	5.69	0.00	0.03
**1435229_at**	GRAM domain containing 1B	Gramd1b	1.54	7.99	0.00	0.01
**1458045_at**	---	---	1.54	5.51	0.00	0.03
**1433902_at**	kelch repeat and BTB (POZ) domain containing 8	Kbtbd8	1.54	5.56	0.00	0.03
**1417876_at**	Fc receptor, IgG, high affinity I	Fcgr1	1.55	6.40	0.00	0.02
**1443728_at**	---	---	1.55	5.29	0.00	0.04
**1421897_at**	ELK1, member of ETS oncogene family	Elk1	1.56	5.37	0.00	0.04
**1436425_at**	KN motif and ankyrin repeat domains 4	Kank4	1.56	5.14	0.00	0.04
**1425373_a_at**	proteasome assembly chaperone 2-like /// proteasome (prosome, macropain) assembly chaperone 2	LOC101056547 /// Psmg2	1.56	6.97	0.00	0.01
**1434993_at**	family with sequence similarity 5, member C	Fam5c	1.56	5.41	0.00	0.03
**1450268_at**	fidgetin	Fign	1.56	6.15	0.00	0.02
**1453768_a_at**	family with sequence similarity 110, member A	Fam110a	1.56	5.10	0.00	0.04
**1422210_at**	forkhead box D3	Foxd3	1.57	8.41	0.00	0.01
**1453303_at**	RIKEN cDNA 4833417J20 gene	4833417J20Rik	1.57	5.44	0.00	0.03
**1449001_at**	isovaleryl coenzyme A dehydrogenase	Ivd	1.57	6.21	0.00	0.02
**1450650_at**	myosin X	Myo10	1.57	6.16	0.00	0.02
**1429185_at**	Rho guanine nucleotide exchange factor (GEF) 26	Arhgef26	1.57	7.58	0.00	0.01
**1455712_at**	histone cluster 3, H2a	Hist3h2a	1.57	8.70	0.00	0.00
**1429324_at**	Shc SH2-domain binding protein 1-like	Shcbp1l	1.57	6.06	0.00	0.02
**1419028_at**	cyclic AMP-regulated phosphoprotein, 21	Arpp21	1.58	6.15	0.00	0.02
**1441107_at**	doublesex and mab-3 related transcription	Dmrta2	1.58	5.36	0.00	0.04

	factor like family A2					
**1438682_at**	phosphatidylinositol 3-kinase, regulatory subunit, polypeptide 1 (p85 alpha)	Pik3r1	1.58	6.62	0.00	0.02
**1417856_at**	avian reticuloendotheliosis viral (v-rel) oncogene related B	Relb	1.58	5.56	0.00	0.03
**1436050_x_at**	hairy and enhancer of split 6	Hes6	1.58	5.70	0.00	0.03
**1432590_at**	RIKEN cDNA 4930573O21 gene	4930573O21Rik	1.58	6.96	0.00	0.01
**1445093_at**	---	---	1.59	5.63	0.00	0.03
**1434513_at**	ATPase type 13A3	Atp13a3	1.59	5.77	0.00	0.03
**1459030_at**	butyrobetaine (gamma), 2-oxoglutarate dioxygenase 1 (gamma-butyrobetaine hydroxylase)	Bbox1	1.59	6.67	0.00	0.01
**1415977_at**	myo-inositol 1-phosphate synthase A1	Isyna1	1.59	8.10	0.00	0.01
**1440624_at**	---	---	1.59	5.39	0.00	0.03
**1458409_at**	---	---	1.59	7.12	0.00	0.01
**1424480_s_at**	thymoma viral proto-oncogene 2	Akt2	1.59	10.31	0.00	0.00
**1459813_at**	RIKEN cDNA 1700012D01 gene /// transmembrane protein 194	1700012D01Rik /// Tmem194	1.59	7.11	0.00	0.01
**1424967_x_at**	troponin T2, cardiac	Tnnt2	1.59	6.16	0.00	0.02
**1451689_a_at**	SRY-box containing gene 10	Sox10	1.59	5.56	0.00	0.03
**1449049_at**	toll-like receptor 1	Tlr1	1.60	5.96	0.00	0.02
**1455646_at**	RIKEN cDNA 2010004M13 gene	2010004M13Rik	1.60	5.64	0.00	0.03
**1435775_at**	circadian locomotor output cycles kaput	Clock	1.60	7.10	0.00	0.01
**1457342_at**	IKAROS family zinc finger 4	Ikzf4	1.60	5.28	0.00	0.04
**1420797_at**	otogelin	Otog	1.61	9.39	0.00	0.00
**1456242_at**	predicted gene 7325	Gm7325	1.61	5.29	0.00	0.04
**1452650_at**	tripartite motif-containing 62	Trim62	1.61	5.23	0.00	0.04
**1449164_at**	CD68 antigen	Cd68	1.61	5.41	0.00	0.03
**1449532_at**	cholinergic receptor, nicotinic, gamma polypeptide	Chrng	1.61	6.18	0.00	0.02

**1421063_s_at**	small nuclear ribonucleoprotein N /// SNRPN upstream reading frame	Snrpn /// Snurf	1.61	5.86	0.00	0.03
**1420955_at**	visinin-like 1	Vsnl1	1.62	7.24	0.00	0.01
**1429060_at**	metastasis associated lung adenocarcinoma transcript 1 (non-coding RNA)	Malat1	1.62	5.30	0.00	0.04
**1460626_at**	septin 11	Sep-11	1.62	5.67	0.00	0.03
**1457063_at**	---	---	1.62	7.21	0.00	0.01
**1423909_at**	transmembrane protein 176A	Tmem176a	1.62	7.30	0.00	0.01
**1460440_at**	latrophilin 3	Lphn3	1.62	5.74	0.00	0.03
**1444550_at**	coiled-coil domain containing 167	Ccdc167	1.63	4.96	0.00	0.05
**1451411_at**	G protein-coupled receptor, family C, group 5, member B	Gprc5b	1.63	7.43	0.00	0.01
**1423071_x_at**	RIKEN cDNA 6720475J19 gene	6720475J19Rik	1.63	8.55	0.00	0.01
**1448212_at**	proteasome assembly chaperone 2-like /// proteasome (prosome, macropain) assembly chaperone 2	LOC101056547 /// Psmg2	1.63	7.46	0.00	0.01
**1438673_at**	solute carrier family 4, sodium bicarbonate cotransporter, member 7	Slc4a7	1.64	6.38	0.00	0.02
**1434709_at**	neuron-glia-CAM-related cell adhesion molecule	Nrcam	1.64	5.69	0.00	0.03
**1434754_at**	RAP1 GTPase activating protein 2	Rap1gap2	1.64	5.45	0.00	0.03
**1417050_at**	C1q and tumor necrosis factor related protein 4	C1qtnf4	1.65	5.57	0.00	0.03
**1442873_at**	fidgetin	Fign	1.65	5.79	0.00	0.03
**1420377_at**	ST8 alpha-N-acetyl-neuraminide alpha-2,8-sialyltransferase 2	St8sia2	1.65	6.38	0.00	0.02
**1421324_a_at**	thymoma viral proto-oncogene 2	Akt2	1.65	5.92	0.00	0.02
**1457732_at**	---	---	1.65	5.52	0.00	0.03
**1420617_at**	cytoplasmic polyadenylation element binding protein 4	Cpeb4	1.66	5.26	0.00	0.04
**1452021_a_at**	hairy and enhancer of split 6	Hes6	1.66	6.47	0.00	0.02

**1442884_at**	hepatocyte growth factor	Hgf	1.66	7.09	0.00	0.01
**1424754_at**	membrane-spanning 4-domains, subfamily A, member 7	Ms4a7	1.67	7.07	0.00	0.01
**1427019_at**	protein tyrosine phosphatase, receptor type Z, polypeptide 1	Ptprz1	1.67	6.14	0.00	0.02
**1453595_at**	RIKEN cDNA 2900064B18 gene	2900064B18Rik	1.67	6.73	0.00	0.01
**1440999_at**	zinc finger protein 697	Zfp697	1.67	6.38	0.00	0.02
**1418204_s_at**	allograft inflammatory factor 1	Aif1	1.68	8.94	0.00	0.00
**1417702_a_at**	histamine N-methyltransferase	Hnmt	1.68	5.97	0.00	0.02
**1444689_at**	WD repeat domain 67	Wdr67	1.68	5.78	0.00	0.03
**1456833_at**	G protein-coupled receptor 17	Gpr17	1.68	6.79	0.00	0.01
**1416225_at**	alcohol dehydrogenase 1 (class I)	Adh1	1.69	5.60	0.00	0.03
**1429214_at**	ADAMTS-like 2	Adamtsl2	1.69	7.64	0.00	0.01
**1455876_at**	solute carrier family 4, sodium bicarbonate cotransporter, member 7	Slc4a7	1.69	7.20	0.00	0.01
**1443332_at**	---	---	1.70	6.93	0.00	0.01
**1442351_a_at**	cDNA sequence BC029214	BC029214	1.70	6.24	0.00	0.02
**1421818_at**	B cell leukemia/lymphoma 6	Bcl6	1.71	6.94	0.00	0.01
**1444516_at**	---	---	1.71	6.97	0.00	0.01
**1420895_at**	transforming growth factor, beta receptor I	Tgfbr1	1.72	10.14	0.00	0.00
**1448823_at**	chemokine (C-X-C motif) ligand 12	Cxcl12	1.72	10.59	0.00	0.00
**1451021_a_at**	Kruppel-like factor 5	Klf5	1.73	6.24	0.00	0.02
**1455766_at**	gamma-aminobutyric acid (GABA) A receptor, subunit alpha 1	Gabra1	1.73	7.07	0.00	0.01
**1425515_at**	phosphatidylinositol 3-kinase, regulatory subunit, polypeptide 1 (p85 alpha)	Pik3r1	1.73	7.86	0.00	0.01
**1418485_at**	solute carrier family 4 (anion exchanger), member 3	Slc4a3	1.74	10.42	0.00	0.00
**1431050_at**	ribosomal protein S6 kinase, polypeptide 5	Rps6ka5	1.74	6.84	0.00	0.01
**1444983_at**	---	---	1.74	5.65	0.00	0.03

**1436758_at**	histone deacetylase 4	Hdac4	1.74	5.29	0.00	0.04
**1433388_at**	RIKEN cDNA 2900022M07 gene	2900022M07Rik	1.74	9.15	0.00	0.00
**1437385_at**	collagen and calcium binding EGF domains 1	Ccbe1	1.74	8.59	0.00	0.01
**1419050_at**	transmembrane protein 8C	Tmem8c	1.74	5.97	0.00	0.02
**1449307_at**	dysbindin (dystrobrevin binding protein 1) domain containing 1	Dbndd1	1.75	5.32	0.00	0.04
**1440343_at**	ribosomal protein S6 kinase, polypeptide 5	Rps6ka5	1.75	7.14	0.00	0.01
**1434025_at**	---	---	1.76	5.86	0.00	0.03
**1458551_at**	---	---	1.76	5.33	0.00	0.04
**1438423_at**	single-stranded DNA binding protein 2	Ssbp2	1.76	5.64	0.00	0.03
**1440759_at**	potassium voltage-gated channel, shaker-related subfamily, member 2	Kcna2	1.77	6.40	0.00	0.02
**1448591_at**	cathepsin S	Ctss	1.77	5.64	0.00	0.03
**1437939_s_at**	cathepsin C	Ctsc	1.78	6.48	0.00	0.02
**1421075_s_at**	cytochrome P450, family 7, subfamily b, polypeptide 1	Cyp7b1	1.78	9.91	0.00	0.00
**1456922_at**	sorting nexin 29	Snx29	1.79	9.35	0.00	0.00
**1417574_at**	chemokine (C-X-C motif) ligand 12	Cxcl12	1.79	6.01	0.00	0.02
**1423213_at**	plexin C1	Plxnc1	1.81	10.65	0.00	0.00
**1442082_at**	complement component 3a receptor 1	C3ar1	1.81	9.10	0.00	0.00
**1439412_at**	---	---	1.82	6.04	0.00	0.02
**1437418_at**	predicted gene 3515	Gm3515	1.82	7.70	0.00	0.01
**1435831_at**	uroplakin 1B	Upk1b	1.82	5.78	0.00	0.03
**1441865_at**	---	---	1.83	6.27	0.00	0.02
**1436912_at**	calcium channel, voltage-dependent, beta 4 subunit	Cacnb4	1.83	5.42	0.00	0.03
**1422694_at**	tweety homolog 1 (Drosophila)	Ttyh1	1.83	6.37	0.00	0.02
**1458052_at**	---	---	1.84	5.46	0.00	0.03
**1423072_at**	RIKEN cDNA 6720475J19 gene	6720475J19Rik	1.85	5.28	0.00	0.04
**1425235_s_at**	collagen, type XX, alpha 1	Col20a1	1.86	7.74	0.00	0.01

**1421074_at**	cytochrome P450, family 7, subfamily b, polypeptide 1	Cyp7b1	1.86	10.67	0.00	0.00
**1419004_s_at**	B cell leukemia/lymphoma 2 related protein A1a /// B cell leukemia/lymphoma 2 related protein A1b /// B cell leukemia/lymphoma 2 related protein A1d	Bcl2a1a /// Bcl2a1b /// Bcl2a1d	1.86	7.38	0.00	0.01
**1458680_at**	hephaestin-like 1	Hephl1	1.86	6.46	0.00	0.02
**1420401_a_at**	receptor (calcitonin) activity modifying protein 3	Ramp3	1.86	8.88	0.00	0.00
**1451453_at**	death-associated protein kinase 2	Dapk2	1.87	6.11	0.00	0.02
**1446720_at**	---	---	1.87	5.00	0.00	0.05
**1427946_s_at**	dihydropyrimidine dehydrogenase	Dpyd	1.88	7.06	0.00	0.01
**1457152_at**	---	---	1.89	6.37	0.00	0.02
**1451537_at**	chitinase 3-like 1	Chi3l1	1.89	10.29	0.00	0.00
**1429273_at**	BMP-binding endothelial regulator	Bmper	1.90	10.95	0.00	0.00
**1429861_at**	protocadherin 9	Pcdh9	1.90	5.23	0.00	0.04
**1456208_at**	glycerol-3-phosphate acyltransferase 2, mitochondrial	Gpat2	1.91	12.65	0.00	0.00
**1451280_at**	cyclic AMP-regulated phosphoprotein, 21	Arpp21	1.91	5.62	0.00	0.03
**1439026_at**	transient receptor potential cation channel, subfamily M, member 3	Trpm3	1.92	8.03	0.00	0.01
**1434458_at**	follistatin	Fst	1.93	5.28	0.00	0.04
**1444071_at**	RIKEN cDNA 9630013A20 gene	9630013A20Rik	1.93	9.17	0.00	0.00
**1417429_at**	flavin containing monooxygenase 1	Fmo1	1.94	7.82	0.00	0.01
**1443138_at**	---	---	1.96	9.10	0.00	0.00
**1421365_at**	follistatin	Fst	1.96	10.25	0.00	0.00
**1428203_at**	gamma-aminobutyric acid (GABA) A receptor, subunit beta 2	Gabrb2	1.97	10.36	0.00	0.00
**1448395_at**	secreted frizzled-related protein 1	Sfrp1	2.00	6.00	0.00	0.02
**1457741_at**	predicted gene 12688	Gm12688	2.00	8.03	0.00	0.01
**1425109_at**	solute carrier family 44, member 3	Slc44a3	2.01	7.06	0.00	0.01

**1454782_at**	brain-specific angiogenesis inhibitor 3	Bai3	2.02	5.28	0.00	0.04
**1418139_at**	doublecortin	Dcx	2.03	5.72	0.00	0.03
**1435424_x_at**	shisa homolog 9 (Xenopus laevis)	Shisa9	2.03	6.87	0.00	0.01
**1442226_at**	sema domain, immunoglobulin domain (Ig), short basic domain, secreted, (semaphorin) 3E	Sema3e	2.06	5.18	0.00	0.04
**1432165_at**	RIKEN cDNA 8430422H06 gene	8430422H06Rik	2.06	7.83	0.00	0.01
**1429685_at**	gamma-aminobutyric acid (GABA) A receptor, subunit beta 2	Gabrb2	2.08	7.54	0.00	0.01
**1419618_at**	butyrobetaine (gamma), 2-oxoglutarate dioxygenase 1 (gamma-butyrobetaine hydroxylase)	Bbox1	2.10	8.36	0.00	0.01
**1434172_at**	cannabinoid receptor 1 (brain)	Cnr1	2.11	7.35	0.00	0.01
**1439872_at**	potassium voltage-gated channel, shaker-related subfamily, member 2	Kcna2	2.11	6.79	0.00	0.01
**1438751_at**	solute carrier family 30, member 10	Slc30a10	2.13	10.73	0.00	0.00
**1456633_at**	transient receptor potential cation channel, subfamily M, member 3	Trpm3	2.13	12.07	0.00	0.00
**1440249_at**	AKNA domain containing 1	Aknad1	2.14	5.89	0.00	0.02
**1440429_at**	cyclic AMP-regulated phosphoprotein, 21	Arpp21	2.14	5.53	0.00	0.03
**1452520_a_at**	cholinergic receptor, nicotinic, gamma polypeptide	Chrng	2.14	7.35	0.00	0.01
**1426914_at**	MARVEL (membrane-associating) domain containing 2	Marveld2	2.15	7.55	0.00	0.01
**1452517_at**	pleckstrin homology domain containing, family H (with MyTH4 domain) member 1	Plekhh1	2.15	8.83	0.00	0.00
**1432466_a_at**	apolipoprotein E	Apoe	2.17	9.00	0.00	0.00
**1450042_at**	aristaless related homeobox	Arx	2.18	6.40	0.00	0.02
**1442121_at**	cyclic AMP-regulated phosphoprotein, 21	Arpp21	2.18	6.02	0.00	0.02
**1448326_a_at**	cellular retinoic acid binding protein I	Crabp1	2.20	11.61	0.00	0.00
**1425236_at**	collagen, type XX, alpha 1	Col20a1	2.21	5.98	0.00	0.02
**1420699_at**	C-type lectin domain family 7, member a	Clec7a	2.23	6.80	0.00	0.01

**1452027_a_at**	transformation related protein 63	Trp63	2.25	8.93	0.00	0.00
**1418852_at**	cholinergic receptor, nicotinic, alpha polypeptide 1 (muscle)	Chrna1	2.28	7.86	0.00	0.01
**1456923_at**	transient receptor potential cation channel, subfamily M, member 3	Trpm3	2.30	5.49	0.00	0.03
**1447040_at**	---	---	2.33	8.10	0.00	0.01
**1430855_at**	collagen, type XX, alpha 1	Col20a1	2.36	10.39	0.00	0.00
**1454693_at**	histone deacetylase 4	Hdac4	2.36	7.73	0.00	0.01
**1460187_at**	secreted frizzled-related protein 1	Sfrp1	2.36	5.26	0.00	0.04
**1460123_at**	G protein-coupled receptor 1	Gpr1	2.37	5.56	0.00	0.03
**1427076_at**	macrophage expressed gene 1	Mpeg1	2.38	7.57	0.00	0.01
**1422573_at**	adenosine monophosphate deaminase 3	Ampd3	2.42	5.07	0.00	0.04
**1450779_at**	fatty acid binding protein 7, brain	Fabp7	2.42	12.05	0.00	0.00
**1450875_at**	G protein-coupled receptor 37	Gpr37	2.54	8.48	0.00	0.01
**1441794_at**	---	---	2.54	6.84	0.00	0.01
**1436889_at**	gamma-aminobutyric acid (GABA) A receptor, subunit alpha 1	Gabra1	2.54	14.99	0.00	0.00
**1451257_at**	acyl-CoA synthetase long-chain family member 6	Acsl6	2.71	7.94	0.00	0.01
**1419905_s_at**	hydroxyprostaglandin dehydrogenase 15 (NAD)	Hpgd	2.76	6.04	0.00	0.02
**1422552_at**	reprimo, TP53 dependent G2 arrest mediator candidate	Rprm	2.81	11.64	0.00	0.00
**1418937_at**	deiodinase, iodothyronine, type II	Dio2	2.87	11.36	0.00	0.00
**1418690_at**	protein tyrosine phosphatase, receptor type Z, polypeptide 1	Ptprz1	3.15	12.87	0.00	0.00
**1445787_at**	coiled-coil domain containing 162	Ccdc162	3.17	7.83	0.00	0.01
**1440878_at**	runt related transcription factor 1	Runx1	3.25	9.23	0.00	0.00
**1452065_at**	V-set and transmembrane domain containing 2A	Vstm2a	3.28	10.55	0.00	0.00
**1435053_s_at**	pleckstrin homology domain containing, family	Plekhh1	3.57	7.62	0.00	0.01

	H (with MyTH4 domain) member 1					
**1418203_at**	phorbol-12-myristate-13-acetate-induced protein 1	Pmaip1	3.69	15.37	0.00	0.00
**1422865_at**	runt related transcription factor 1	Runx1	3.94	11.52	0.00	0.00
**1422864_at**	runt related transcription factor 1	Runx1	4.08	12.89	0.00	0.00
**1447807_s_at**	pleckstrin homology domain containing, family H (with MyTH4 domain) member 1	Plekhh1	4.52	11.57	0.00	0.00
**1451203_at**	myoglobin	Mb	4.75	11.87	0.00	0.00
**1456953_at**	collagen, type XIX, alpha 1	Col19a1	5.11	10.54	0.00	0.00
**1421698_a_at**	collagen, type XIX, alpha 1	Col19a1	5.13	9.40	0.00	0.00
**1438059_at**	cortexin 3	Ctxn3	5.23	6.29	0.00	0.02
**1440085_at**	ectodysplasin A2 receptor	Eda2r	5.73	8.76	0.00	0.00
**1438540_at**	collagen, type XXV, alpha 1	Col25a1	6.51	9.54	0.00	0.00

Supplementary Table S2: The 10 most significantly regulated GO categories for the downregulated and upregulated DEGs according to a *DAVID* GO enrichment analysis for biological process, cellular compartment and molecular function.

Category	Term	Count	P Value	Genes
		**Downregulated DEGs**		
**GOTERM_CC_5**	GO:0030016~myofibril	6	7.16E-04	MYL2, NRAP, KRT8, SMTNL1, ANKRD1, CSRP3
**GOTERM_CC_5**	GO:0043292~contractile fiber	6	8.70E-04	MYL2, NRAP, KRT8, SMTNL1, ANKRD1, CSRP3
**GOTERM_CC_5**	GO:0031674~I band	4	0.008124	KRT8, SMTNL1, ANKRD1, CSRP3
**GOTERM_MF_5**	GO:0005540~hyaluronic acid binding	3	0.009505	TNFAIP6, LYVE1, CD44
**GOTERM_CC_5**	GO:0005667~transcription factor complex	7	0.009964	FOS, SNAI3, ATF3, JUN, PDLIM1, ANKRD1, NPAS4
**GOTERM_BP_5**	GO:0009888~tissue development	12	0.011771	FGF6, CAV2, MYL2, CD44, IRF6, JUN, TNC, TNFRSF19, SEMA3C, TGFB1I1, CSRP3, GAP43
**GOTERM_BP_5**	GO:0001936~regulation of endothelial cell proliferation	3	0.011776	WNT2, CAV2, VASH1
**GOTERM_BP_5**	GO:0007517~muscle organ	6	0.012796	CAV2, MYL2, TNC, SMTNL1, TAGLN2, CSRP3

	development			
**GOTERM_BP_5**	GO:0016055~Wnt receptor signaling pathway	5	0.019551	WNT2, FZD10, CCDC88C, SFRP4, TGFB1I1
**GOTERM_MF_5**	GO:0005509~calcium ion binding	13	0.020465	CD97, PTX4, RASGRP3, MYL2, MYL3, PCDH20, FBN1, RASGRP2, PADI4, SPON2, ANXA3, MYL9, THBS4
		**Upregulated DEGs**		
**GOTERM_BP_5**	GO:0042981~regulation of apoptosis	16	3.03E-05	TGFBR1, MALT1, HGF, PMAIP1, DAPK2, FCGR1, CDKN1A, BCL2A1D, PSMG2, BCL2A1B, BCL2A1A, APOE, TRP63, BCL6, BMF, PIK3R1
**GOTERM_BP_5**	GO:0043067~regulation of programmed cell death	16	3.51E-05	TGFBR1, MALT1, HGF, PMAIP1, DAPK2, FCGR1, CDKN1A, BCL2A1D, PSMG2, BCL2A1B, BCL2A1A, APOE, TRP63, BCL6, BMF, PIK3R1
**GOTERM_CC_5**	GO:0034702~ion channel complex	7	0.001276	GABRA1, GABRB2, KCNA2, TTYH1, CACNB4, CHRNA1, CHRNG
**GOTERM_BP_5**	GO:0002768~immune response-regulating cell surface receptor signaling pathway	4	0.00516	BCL2A1D, MALT1, CACNB4, CLEC7A
**GOTERM_BP_5**	GO:0002757~immune response-activating signal transduction	4	0.006585	BCL2A1D, MALT1, CACNB4, CLEC7A
**GOTERM_BP_5**	GO:0002764~immune response-regulating signal transduction	4	0.008224	BCL2A1D, MALT1, CACNB4, CLEC7A
**GOTERM_BP_5**	GO:0022008~neurogenesis	11	0.010499	ARX, NRCAM, SOX10, PTPRZ1, TGFBR1, NTRK2, RUNX1, FABP7, DCX, CXCL12, ACSL6
**GOTERM_BP_5**	GO:0012502~induction of programmed cell death	6	0.010809	APOE, TGFBR1, TRP63, PMAIP1, DAPK2, FCGR1
**GOTERM_BP_5**	GO:0043066~negative regulation of apoptosis	7	0.012182	CDKN1A, BCL2A1A, TGFBR1, TRP63, BCL6, HGF, PIK3R1
**GOTERM_BP_5**	GO:0043069~negative regulation of programmed cell death	7	0.013382	CDKN1A, BCL2A1A, TGFBR1, TRP63, BCL6, HGF, PIK3R1

Supplementary Table S3: Supplementary references.

№	Reference
**S1.**	Ulke-Lemee, A., et al., *Two domains of the smoothelin-like 1 protein bind apo- and calcium-calmodulin independently.* Biochim Biophys Acta, 2014. **1844**(9): p. 1580-90.
**S2.**	Winder, S.J., et al., *Calponin-calmodulin interaction: properties and effects on smooth and skeletal muscle actin binding and actomyosin ATPases.* Biochemistry, 1993. **32**(48): p. 13327-33.
**S3.**	Miller, M.K., et al., *The muscle ankyrin repeat proteins: CARP, ankrd2/Arpp and DARP as a family of titin filament-based stress response molecules.* J Mol Biol, 2003. **333**(5): p. 951-64.
**S4.**	Ursitti, J.A., et al., *Cloning and characterization of cytokeratins 8 and 19 in adult rat striated muscle. Interaction with the dystrophin glycoprotein complex.* J Biol Chem, 2004. **279**(40): p. 41830-8.
**S5.**	Knoll, R., B. Buyandelger, and M. Lab, *The sarcomeric Z-disc and Z-discopathies.* J Biomed Biotechnol, 2011. **2011**: p. 569628.
**S6.**	Marotta, M., et al., *Muscle genome-wide expression profiling during disease evolution in mdx mice.* Physiol Genomics, 2009. **37**(2): p. 119-32.
**S7.**	Hijikata, T., et al., *Plectin 1 links intermediate filaments to costameric sarcolemma through beta-synemin, alpha-dystrobrevin and actin.* J Cell Sci, 2008. **121**(Pt 12): p. 2062-74.
**S8.**	Okamoto, O. and S. Fujiwara, *Dermatopontin, a novel player in the biology of the extracellular matrix.* Connect Tissue Res, 2006. **47**(4): p. 177-89.
**S9.**	Llano-Diez, M., et al., *Muscle wasting and the temporal gene expression pattern in a novel rat intensive care unit model.* BMC Genomics, 2011. **12**: p. 602.
**S10.**	Voermans, N.C., et al., *Compound heterozygous mutations of the TNXB gene cause primary myopathy.* Neuromuscul Disord, 2014. **24**(1): p. 88-9.
**S11.**	Mackey, A.L., et al., *Sequenced response of extracellular matrix deadhesion and fibrotic regulators after muscle damage is involved in protection against future injury in human skeletal muscle.* FASEB J, 2011. **25**(6): p. 1943-59.
**S12.**	Lambeir, A.M., et al., *Dipeptidyl-peptidase IV from bench to bedside: an update on structural properties, functions, and clinical aspects of the enzyme DPP IV.* Crit Rev Clin Lab Sci, 2003. **40**(3): p. 209-94.
**S13.**	Mylona, E., et al., *CD44 regulates myoblast migration and differentiation.* J Cell Physiol, 2006. **209**(2): p. 314-21.
**S14.**	Shapland, C., et al., *Purification and properties of transgelin: a transformation and shape change sensitive actin-gelling protein.* J Cell Biol, 1993. **121**(5): p. 1065-73.
**S15.**	Goriounov, D., C.L. Leung, and R.K. Liem, *Protein products of human Gas2-related genes on chromosomes 17 and 22 (hGAR17 and hGAR22) associate with both microfilaments and microtubules.* J Cell Sci, 2003. **116**(Pt 6): p. 1045-58.
**S16.**	Sejersen, T. and U. Lendahl, *Transient expression of the intermediate filament nestin during skeletal muscle development.* J Cell Sci, 1993. **106 (Pt 4)**: p. 1291-300.
**S17.**	Gabriel, L.A., et al., *ADAMTSL4, a secreted glycoprotein widely distributed in the eye, binds fibrillin-1 microfibrils and accelerates*

	microfibril biogenesis. Invest Ophthalmol Vis Sci, 2012. **53**(1): p. 461-9.
**S18.**	Zhu, Y., et al., *Kank proteins: a new family of ankyrin-repeat domain-containing proteins.* Biochim Biophys Acta, 2008. **1780**(2): p. 128-33.
**S19.**	Kostrominova, T.Y., et al., *Comparison of gene expression of 2-mo denervated, 2-mo stimulated-denervated, and control rat skeletal muscles.* Physiol Genomics, 2005. **22**(2): p. 227-43.
**S20.**	Autieri, M.V., S.E. Kelemen, and K.W. Wendt, *AIF-1 is an actin-polymerizing and Rac1-activating protein that promotes vascular smooth muscle cell migration.* Circ Res, 2003. **92**(10): p. 1107-14.
**S21.**	Millay, D.P., et al., *Myomaker is a membrane activator of myoblast fusion and muscle formation.* Nature, 2013. **499**(7458): p. 301-5.
**S22.**	Ogawa, R., et al., *Doublecortin marks a new population of transiently amplifying muscle progenitor cells and is required for myofiber maturation during skeletal muscle regeneration.* Development, 2015. **142**(1): p. 51-61.
**S23.**	Sumiyoshi, H., et al., *Embryonic expression of type XIX collagen is transient and confined to muscle cells.* Dev Dyn, 2001. **220**(2): p. 155-62.
**S24.**	Tanaka, T., et al., *CLAC-P/collagen type XXV is required for the intramuscular innervation of motoneurons during neuromuscular development.* J Neurosci, 2014. **34**(4): p. 1370-9.
